# Overexpression of *OsAGO1b* Induces Adaxially Rolled Leaves by Affecting Leaf Abaxial Sclerenchymatous Cell Development in Rice

**DOI:** 10.1186/s12284-019-0323-9

**Published:** 2019-08-08

**Authors:** Youhan Li, Yiqi Yang, Ye Liu, Dexia Li, Yahuan Zhao, Zhijie Li, Ying Liu, Dagang Jiang, Jing Li, Hai Zhou, Jianghua Chen, Chuxiong Zhuang, Zhenlan Liu

**Affiliations:** 1 State Key Laboratory for Conservation and Utilization of Subtropical Agro-bioresources, College of Life Sciences, South China Agricultural University, Guangzhou, 510642 China; 2 Key Laboratory of Tropical Plant Resources and Sustainable Use, CAS Center for Excellence in Molecular Plant Sciences, Xishuangbanna Tropical Botanical Garden, Chinese Academy of Sciences, Kunming, 650223 China; 3 School of Life Sciences, University of Science and Technology of China, Hefei, 230026 China; 4 Guangdong Key Laboratory for Innovative Development and Utilization of Forest Plant Germplasm, South China Agricultural University, Guangzhou, 510642 China

**Keywords:** ARGONAUTE protein, Leaf rolling, *OsAGO1b*, Rice, Sclerenchymatous cell, Small RNA

## Abstract

**Background:**

ARGONAUTE 1 (AGO1) proteins can recruit small RNAs to regulate gene expression, involving several growth and development processes in *Arabidopsis*. Rice genome contains four *AGO1* genes, *OsAGO1a* to *OsAGO1d*. However, the regulatory functions to rice growth and development of each *AGO1* gene are still unknown.

**Results:**

We obtained overexpression and RNAi transgenic lines of each *OsAGO1* gene. However, only up- and down-regulation of *OsAGO1b* caused multiple abnormal phenotypic changes in rice, indicating that *OsAGO1b* is the key player in rice growth and organ development compared with other three *OsAGO1s*. qRT-PCR assays showed that *OsAGO1b* was almost unanimously expressed in leaves at different developmental stages, and strongly expressed in spikelets at S1 to S3 stages. OsAGO1b is a typical AGO protein, and co-localized in both the nucleus and cytoplasm simultaneously. Overexpression of *OsAGO1b* caused adaxially rolled leaves and a series of abnormal phenotypes, such as the reduced tiller number and plant height. Knockdown lines of *OsAGO1b* showed almost normal leaves, but the seed setting percentage was significantly reduced accompanied by the disturbed anther patterning and reduced pollen fertility. Further anatomical observation revealed that *OsAGO1b* overexpression plants showed the partially defective development of sclerenchymatous cells on the abaxial side of leaves. In situ hybridization showed *OsAGO1b* mRNA was uniformly accumulated in P1 to P3 primordia without polarity property, suggesting *OsAGO1b* did not regulate the adaxial-abaxial polarity development directly. The expression levels of several genes related to leaf polarity development and vascular bundle differentiation were observably changed. Notably, the accumulation of *miR166* and *TAS3-siRNA* was decreased, and their targeted *OSHBs* and *OsARFs* were significantly up-regulated. The mRNA distribution patterns of *OSHB3* and *OsARF4* in leaves remained almost unchanged between ZH11 and *OsAGO1b* overexpression lines, but their expression levels were enhanced at the regions of vascular bundles and sclerenchymatous cell differentiation.

**Conclusions:**

In summary, we demonstrated *OsAGO1b* is the leading player among four *OsAGO1s* in rice growth and development. We propose that *OsAGO1b* may regulate the abaxial sclerenchymatous cell differentiation by affecting the expression of *OSHBs* in rice.

**Electronic supplementary material:**

The online version of this article (10.1186/s12284-019-0323-9) contains supplementary material, which is available to authorized users.

## Background

The leaf is a type of lateral organ of seed plants, formed from the flanks of the shoot apical meristem (SAM) (Bowman et al. 2002). In order to improve the biomass and grain yield of crops, the plants should have more net photosynthesis. Leaf is the main organ to process photosynthesis in rice, and it is necessary to increase the photosynthetic rates at leaf level (Horton 2000). It is widely accepted that the most important leaf morphological features of high-yielding rice should be long, erect, narrow, V-type (rolled leaves) and thick (Yuan 1997). Rice plants with suitable inwardly rolled leaves can improve light transmission and increase the light capture of lower leaves for photosynthesis and nitrogen storage for grain filling (Sinclair and Sheehy 1999; Sakamoto et al. 2006).

At the initiation stage of leaf formation, three polarity axes, i.e. adaxial-abaxial, medial-lateral and proximal-distal, of the leaf primordia are established on the flanks of SAM (Bowman et al. 2002; Kidner and Timmermans 2007; Braybrook and Kuhlemeier 2010; Moon and Hake 2011). Generally, plants show two types of transversely rolled leaves: inward/adaxial and outward/abaxial rolling. The rolled leaf formation is principally regulated by adaxial-abaxial polarity development (Yamaguchi et al. 2012). The molecular mechanism of leaf polarity establishment has been extensively studied in *Arabidopsis thaliana*. *PHABULOSA* (*PHB*), *PHAVOLUTA* (*PHV*), and *REVOLUTE* (*REV*) belong to the *HD-ZIP III* gene family, encoding class III homeodomain-leucine zipper transcription factors that regulate adaxial cell development in leaves (McConnell et al. 2001; Prigge et al. 2005; Otsuga et al. 2008). *KANADI* (*KAN*) encodes a GARP transcription factor regulating leaf abaxial identity (Kerstetter et al. 2001). *YABBY2* and *YABBY3*, encoding YABBY transcription factors, are expressed on the abaxial side of leaf primordia and have been proposed to specify the abaxial cell fate of leaves (Siegfried et al. 1999). A few small RNAs have also been proved to regulate leaf polarity development. *HD-ZIP III* genes, such as *PHB* and *PHV*, are the targets of miR165/166 that can affect leaf adaxial-abaxial polarity development by repressing *HD-ZIP III* genes (Reinhart et al. 2002; Williams et al. 2005b). *TAS3* trans-acting small interference RNAs (tasiRNAs) can bind to *AUXIN RESPONSE FACTOR 3*/*ETTIN* (*ARF3*/*ETT*) that determines the abaxial cell fate to regulate leaf polarity identity (Garcia et al. 2006).

In rice, a number of genes regulating leaf rolling have been cloned and characterized. Among them, several genes regulate leaf rolling through affecting bulliform cell development (Zou et al. 2011; Xiang et al. 2012; Chen et al. 2015; Yang et al. 2016; Liang et al. 2018) or cell wall component (Li et al. 2009; Hu et al. 2010; Yang et al. 2014b; Ye et al. 2015). Moreover, *SHALLOT-LIKE 1* (*SLL1*) and *Semi-Rolled Leaf 2* (*SRL2*)/*NARROW AND ROLLED LEAF 2* (*NRL2*) can promote abaxial sclerenchymatous cell formation to regulate leaf development. *SLL1* encodes a SHAQKYF class MYB transcription factor, homologous to *KAN* in *Arabidopsis*. Its knock-out mutant *sll1* exhibits adaxially rolled leaves with defective sclerenchymatous cell formation caused by deficient programmed cell death (PCD) of abaxial mesophyll cells; *SLL1* overexpression inhibited the development of bulliform cells and sclerenchymatous cells on the adaxial side of leaves (Zhang et al. 2009). *SRL2*/*NRL2* encodes a novel plant-specific protein with an unknown function, and its deficient mutant *srl2* also exhibits adaxially rolled leaves with defective sclerenchymatous cells on the abaxial side; phenotypic observation and gene expression analyses of the *sll1 srl2* double mutant suggested that SLL1 and SRL2 might regulate abaxial cell development in distinct pathways (Liu et al. 2016; Zhao et al. 2016).

ARGONAUTE (AGO) protein members are central components of the RNA-induced silencing complex (RISC) and the key players in the small RNA-mediated gene-silencing pathway (Mallory and Vaucheret 2010; Fang and Qi 2016). Generally, small RNAs are recruited by AGO, and then RISC is guided by small RNA to bind to its target, causing mRNA degradation, translational repression or epigenetic modifications (Baulcombe 2004). It is well known that AGO proteins are involved in many biological processes and participate in the regulation of plant growth and development. The rice genome contains 19 AGO members that can be divided into four groups: MEL1, AGO1, AGO4 and AGO7 (Wu et al. 2009). The biological function of a few rice AGO members were well studied. For instance, MEL1 can bind to the phased small interfering RNAs (phasiRNAs) and is also involved in histone H3 modification of meiotic chromosomes to regulate cell division of the premeiotic germ cells (Nonomura et al. 2007; Komiya et al. 2014; Liu and Nonomura 2016). *OsPNH1* is homologous to *Arabidopsis PINHEAD*/*ZWILLE* (*PNH/ZLL*)/*AGO10* that plays essential roles in SAM formation and leaf adaxial cell specification (Nishimura et al. 2002). *OsAGO7*/*SHOOTLESS 4* (*SHL4*)/*SHOOT ORGANIZATION2* (*SHO2*) involves in tasiRNA biosynthesis, coordinating leaf polarity development (Nagasaki et al. 2007; Itoh et al. 2008b), and *OsAGO7* overexpression causes erect and adaxially rolled leaves (Shi et al. 2007). OsAGO2 participates in the regulation of rice pollen development by epigenetically regulating *OsHXK1* expression (Zheng et al. 2019). OsAGO18 can confer broad-spectrum virus resistance in rice (Du et al. 2011; Wu et al. 2015; Wu et al. 2017). The rice *AGO1* family contains four members: *OsAGO1a*, *OsAGO1b*, *OsAGO1c* and *OsAGO1d*. Transgenic plants with decreased expression of four *AGO1*s simultaneously displayed pleiotropic developmental changes accompanying increased expression of miRNA targets (Wu et al. 2009). In addition, the subset of miRNAs recruited by OsAGO1a, OsAGO1b, and OsAGO1c is different, although most of the miRNAs are almost evenly distributed in the three AGO1 complexes, which suggests that rice AGO1 members might be functionally redundant yet distinct (Wu et al. 2009).

To characterize the biological function of individual *AGO1* in rice, we generated RNAi lines and overexpression lines for each *AGO1* gene. We found that *OsAGO1b* knockdown plants mainly showed severe development defects in anthers, accompanying with the decreased rates of pollen fertility and seed setting. *OsAGO1b*-overexpression lines (OE-*AGO1b*) displayed pleiotropic phenotypes, such as adaxially rolled leaves and significantly decreased tiller numbers, which are distinct from other transgenic lines of *OsAGO1* genes. Further investigation showed that the abaxial sclerenchymatous cells were partially defective in OE-*AGO1b* leaves. The expression levels of some genes and small RNAs related to leaf development such as *OSHBs* and *OsARF* genes were significantly changed in OE-*AGO1b* plants, suggesting a regulatory role of OsAGO1b in leaf development in rice.

## Methods

### Plant Materials and Phenotypic Statistics

Rice (*Oryza sativa* L.) *japonica* variety Zhonghua11 (ZH11) was used as the wild type (WT) control. Two *japonica* rice varieties, Nipponbare (Nip) and Songgeng (Sg), and two *indica* rice varieties, Huanghuazhan (HHZ) and Annong (AN), were also used for rice transformation. WT and transgenic rice lines were grown in the greenhouse at South China Agricultural University, Guangzhou, China. The leaf rolling index (LRI) and leaf erect index (LEI) of the top three leaves were determined at the grain-filling stage as described previously (Shi et al. 2007; Zhang et al. 2009). For testing the LRI, the leaf greatest width of natural state (Lnw) and flat state (Lfw) were measured at the same position. For testing the LEI, the leaf length (from the lamina joint to leaf tip) of natural state (Lnl) and straighten state (Lsl) were measured. The LEI and LRI were calculated as following formulas: LRI (%) = (Lfw-Lnw)/Lfw × 100% and LEI (%) = Lnl/Lsl × 100%, respectively. All the phenotypic data shown in this study were measured using ZH11 and two or three independent transgenic lines, and ten individual plants per line were used for the measurement. The information of primers used in this study was listed in Additional file 1: Table S1.

### Construction of Vectors, Rice Transformation and Southern Blot

The cloning vector pBluescript II SK (+) (pSK) and binary vectors pCAMBIA1380, pCAMBIA1390, pRNAi-Ubi and pOx were used to generate overexpression and RNAi constructs of rice four *AGO1s*. The pOx vector was modified from pRNAi-Ubi and can be used to generate the overexpression construct driven by the maize *Ubiqutin* (*Ubi*) promoter (Hu and Liu 2006). A 1986-bp fragment of the miaze *Ubi* promoter (NCBI accession number: S94464) was amplified from maize genomic DNA and then inserted into pSK, and the resulting construct pSK-*Ubi* was used to provide the *Ubi* constitutive promoter. For *OsAGO1b* overexpression vector construction, the *Ubi* promoter was cut from pSK-*Ubi* and then inserted into pCAMBIA1380, and the resulting construct was named pCAMBIA1380-*Ubi*. A 3399-bp fragment of *OsAGO1b* was amplified from ZH11 cDNA by RT-PCR and was then inserted into pCAMBIA1380-*Ubi* to generate the overexpression construct. For *OsAGO1b* RNAi vector construction, a 191-bp fragment was amplified from ZH11 cDNA by RT-PCR and cloned into pRNAi-Ubi by two orientations. The detailed information for all overexpression and RNAi vector construction of rice four *AGO1s* was listed in Additional file 2: Table S2. Each construct was transformed into rice calli via the *Agrobacterium-*mediated protocol (Hiei et al. 1994). To determine the copy number of T-DNA insertion in transgenic plants, Southern hybridization was performed by using a DIG High Prime DNA Labeling and Detection Starter Kit II (Roche). A 750-bp fragment of the selectable marker gene *hygromycin phosphotransferase* (*Hyg*) was used as the probe. Approximate 7 μg genomic DNA of each sample was digested by *Hin*d III, and the following electrophoresis, blotting, hybridization and signal detection were performed according to the manufacturer’s protocol.

### Phylogenetic Analysis

The protein sequences of 18 AGO1 proteins from different plant species were obtained through searching the GenBank database (https://www.ncbi.nlm.nih.gov/genbank/) of NCBI (https://www.ncbi.nlm.nih.gov/) through blastp using OsAGO1b (Q7XSA2) as a query. The detailed information of protein names, accession numbers and plant species was listed in Additional file 3: Table S3. The multiple alignments of the sequences were performed by ClustalW (Thompson et al. 1994). A phylogenetic tree was constructed using the neighbor-joining method (Saitou and Nei 1987) with 1000 replicates in MEGA 7.0 software (Felsenstein 1985; Kumar et al. 2016).

### Nucleic Acid Extraction and Quantitative Real-Time RT-PCR (qRT-PCR)

Genomic DNA was extracted from expanded leaves by CTAB method. Total RNAs were extracted from different rice tissues using TRIzol Regeant (Life Technologies). Spikelets at different developmental stages were collected according to Zhang and Wilson (2009). RNA samples were treated with DNaseI (New England Biolabs) before reverse transcription, and then single-stranded cDNA was synthesized from 500 ng of total RNA using HiScript II Q RT SuperMix for qPCR (Vazyme Biotech, China). For miRNA and siRNA quantitative expression analysis, stem-loop reverse transcription was performed using stem-loop primers for different small RNAs (Shen et al. 2010). The *OsActin1* (XM_015774830) and the 5S ribosomal RNA gene *rrn5* (NC_011033.1, 282,532..282653, complement) were used as internal standards to normalize the expression levels of detected genes and small RNAs respectively. qRT-PCR was performed using Bio-Rad CFX96 and the 2 × RealStar Power SYBR Mixture (GeneStar, China). The amplification conditions were as follows: 95 °C for 3 min, following 39 cycles of 95 °C for 10 s, 58–60 °C for 10 s, and 72 °C for 10 s. Relative expression levels of genes were measured using the 2^−ΔΔ*C*^_T_ method (Livak and Schmittgen 2001).

### Subcellular Localization of OsAGO1b

The cauliflower mosaic virus (CaMV) *35S* promoter, the *yellow florescence protein* (*YFP*) fragment, and the *nopaline synthase* (*NOS*) terminator of *Agrobacterium tumefaciens* were successively cloned into pUC18 to construct the transient expression vector p35S-YFP as described previously (Yang et al. 2014a). The fragment containing the entire *OsAGO1b* coding region without a start codon was amplified by RT-PCR and then inserted into p35S-YFP, downstream of *YFP*, to form the p35S-YFP-OsAGO1b fusion. The resultant vector was transformed into rice protoplasts prepared from leaf sheaths of 14-day-old seedlings, and transient expression was analyzed as described (Yang et al. 2014a).

### Western Blot Analysis

The amino acid sequence of OsAGO1b (Q7XSA2) was used for epitope design. Seven peptide-affinity polyclonal antibodies against OsAGO1b (amino acids 6–15, 31–40, 48–57, 112–121, 133–142, 164–173, 194–203) were raised in rabbits by Abmart (Shanghai, China). The specificity of the purified antibodies was confirmed by Western blot analysis. For OsAGO1b detection, flag leaves of ZH11 and the transgenic plants at the heading stage were used. Protein extraction, SDS-PAGE and Western blotting were carried out as described previously (Li et al. 2011). Coomassie blue staining was used as a loading control. For OsAGO1b subcellular localization, total, nuclear, and cytoplasmic proteins were extracted from 14-day-old seedlings of ZH11 and fractionated as described previously (Roccaro and Somssich 2011; Wang et al. 2011b). As quality controls for the fractionation, RNase Z (*Os02g0214300*) was used as the cytoplasmic marker (Zhou et al. 2014), and histone H3 (Millipore) was used as the nuclear marker (Ye et al. 2012). Western blot analysis was performed as described previously (Li et al. 2011).

### Anatomical Observation of Leaves

Free-hand and semi-thin transverse sections of leaves were used for anatomical observation. For semi-thin sections, fully expanded leaves of 30-day-old seedlings were harvested and fixed in paraformaldehyde buffer containing 4% (w/v) paraformaldehyde, 0.1 mmol/L sodium phosphate (pH 7.0), 0.25% (v/v) glutaraldehyde and 0.1% (v/v) Tween-20. After fixation, the samples were dehydrated in a series of increasing concentrations of ethanol and were then infiltrated and embedded in Epon-812 resin. The polymerization reaction was performed at 60 °C overnight. Semi-thin sections with a thickness of 2 μm were prepared using a Leica UCT ultramicrotome. The sample sections were stained with toluidine blue O, and then examined and photographed with a ZEISS microscope (Axio Observer D1). For autofluorescence analysis, fully expanded leaves of 30-day-old seedlings were fixed with FAA solution overnight, and were then dehydrated through a series of graded ethanol solutions. After dehydration, the samples were embedded in paraffin, and sectioned at a thickness of 8 μm. For observation of spontaneous fluorescence, the sections were examined and photographed under the excitation light for green florescence protein (GFP) on a ZEISS microscope (Axio Observer D1).

### In Situ Hybridization

The shoot base about 1 cm in length including SAM of 14-day-old seedlings of ZH11 and two *OsAGO1b* overexpression lines were collected and then fixed in FAA overnight. After dehydration by a series of graded ethanol solutions, the samples were embedded in paraffin. Before hybridization, sections with a thickness of 8 μm were prepared. The 522-bp, 738-bp, and 705-bp fragments of the *OsAGO1b*, *OSHB3* and *OsARF4* cDNA respectively were used as probe sequences. The probe sequences were amplified by RT-PCR and then cloned into the pGEM™-T Easy vector (Promega). The resulting recombinant clones were used as templates to amplify the sense and antisense probe sequences followed by in vitro transcription. Probe labeling was performed using the DIG RNA Labeling Kit (Roche), and the hybridization was performed as described previously (Itoh et al. 2008a; Chen et al. 2010).

## Results

### Overexpression of *OsAGO1b* Causes Adaxially Rolled Leaves and Multiple Developmental Defects

**Fig. 1:**
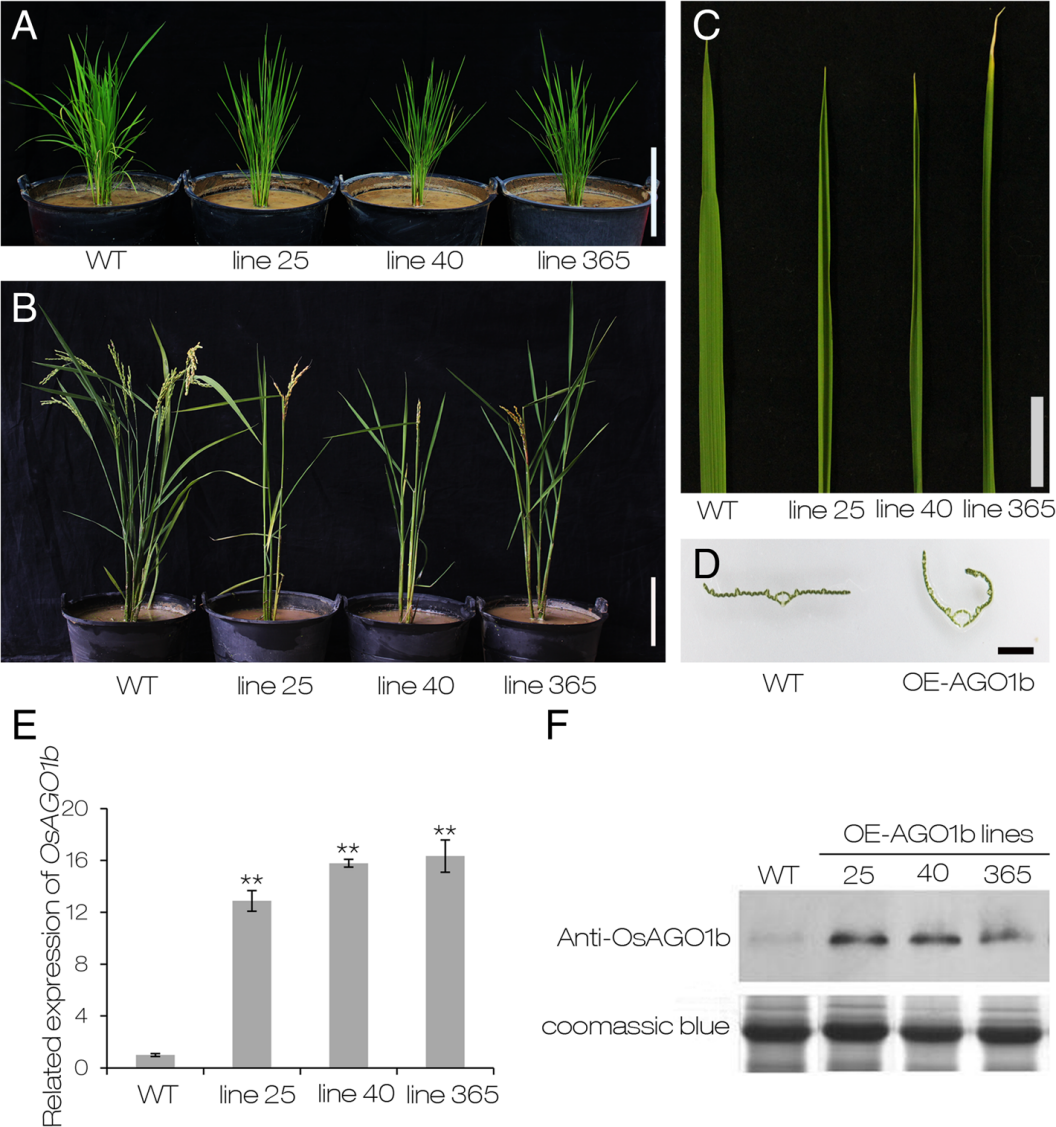
Morphologies and expression analyses of *OsAGO1b* overexpression lines. **a** Morphologies of 30-day-old seedlings of wild type Zhonghua 11 (WT) and *OsAGO1b* overexpression (OE-*AGO1b*) lines (line 25, line 40, line 365). Scale bar = 20 cm. **b** Plant morphologies of WT and OE-*AGO1b* lines at the yellow mature stage. Scale bar = 15 cm. **c** Leaves of 30-day-old seedlings of the WT and OE-*AGO1b* lines. Scale bar = 2 cm. **d** Free-hand cross sections of WT and OE-*AGO1b* leaves from 30-day-old seedlings. Scale bar = 1 mm. **e** Relative expression of *OsAGO1b* in WT and OE-*AGO1b* lines based on qRT-PCR. Error bars represent standard deviations among replicates. The *OsActin1* (XM_015774830) was used as an internal standard to normalize the expression levels of detected genes. **f** Western blot analysis of the OsAGO1b protein in WT and OE-*AGO1b* plants. Coomassic blue staining was used as a loading control. Expanded leaves at the heading stage were used for RNA and protein extraction for (**e**) and (**f**). ** *P* < 0.01 (one-way ANOVA)

**Fig. 2:**
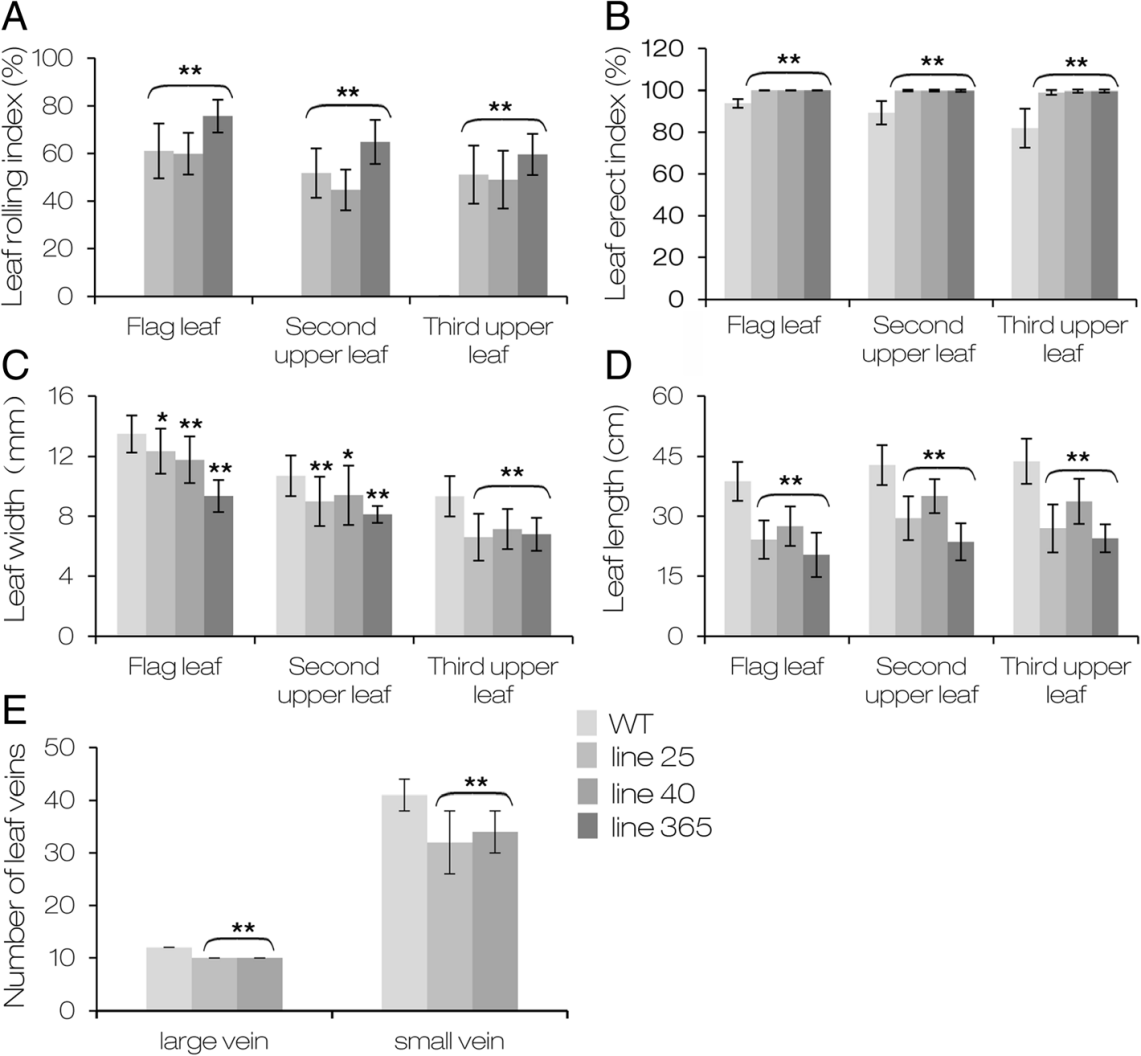
Characteristics of wild type Zhonghua 11 and OE-*AGO1b* leaves. **a**, **b** Leaf rolling index (LRI) and leaf erect index (LEI) of ZH11 (WT) and OE-*AGO1b* (line 25, line 40, line 365) leaves. **c**, **d** Leaf width and leaf length of WT and OE-*AGO1b* leaves. **e** Number of leaf veins of WT and OE-*AGO1b* leaves. Flag leaves at the grain-filling period were used. Results are shown as the mean ± SD in (**a**)-(**e**) (*n* = 10). * *P* < 0.05, ** *P* < 0.01 (one-way ANOVA)

Plant AGO proteins play important roles in the small RNA pathway, as well as in the regulation of various aspects of plant growth and development (Fang and Qi 2016). In the process of characterizing the biological function of individual rice *AGO1* member, transgenic plants overexpressing *OsAGO1b* (OE-*AGO1b*) were obtained. Phenotypic observation showed that most of the T_0_ transgenic seedlings displayed adaxially rolled leaves compared with the seedlings from a control transformation of the empty vector (Additional file 4: Figure S1A). The homozygotes of the three lines (25, 40, and 365) with a single T-DNA insertion confirmed by Southern blot analysis were obtained by planting and hygromycin-resistant selection, and were used for further studies (Additional file 4: Figure S1B). Further observation showed that OE-*AGO1b* plants displayed adaxially rolled leaves from the fourth-leaf stage throughout the whole growth period compared with the wild type ZH11 plants (Fig. 1 a-d; Additional file 5: Figure S2). The expression levels of *OsAGO1b* detected by qRT-PCR were increased by approximately 12- to 15-fold in the three OE-*AGO1b* lines (Fig. 1e), and the accumulation of OsAGO1b protein was also significantly increased (Fig. 1f). At the grain-filling period, the top three leaves of ZH11 were flat, and the LRIs and LEIs of the top three leaves of OE-*AGO1b* plants were significantly increased (Fig. 2 a and b). In addition, the width and length of OE*-AGO1b* leaves were significantly decreased (Fig. 2c and d), and the reduced numbers of veins may have accounted for the narrow leaves of the OE*-AGO1b* plants (Fig. 2e).

OE-*AGO1b* plants also exhibited other phenotypic changes, including significantly decreased tiller numbers, primary and secondary branch numbers of the inflorescence, plant height, inflorescence length, grain numbers and seed setting percentages (Additional file 6: Figure S3). Furthermore, to verify the conservation of *OsAGO1b* regulatory function between *japonica* and *indica* rice, we introduced the OE-*AGO1b* construct into two other *japonica* rice varieties, Nipponbare and Songgeng, as well as into two *indica* rice varieties, Huanghuazhan and Annong. The obtained homozygous plants with increased *OsAGO1b* expression showed almost the same phenotypic changes such as adaxially rolled leaves and reduced tiller numbers as in the ZH11 OE-*AGO1b* lines (Additional file 7: Figure S4). The results mentioned above showed that overexpression of *OsAGO1b* could cause adaxially rolled leaves and accompanied by a series of growth and development defects in rice. Furthermore, the regulatory function of *OsAGO1b* was conserved in *indica* and *japonica* rice.

### *OsAGO1b* Encodes a Typical AGO Protein Which Co-localized in Both the Nucleus and Cytoplasm Simultaneously

**Fig. 3:**
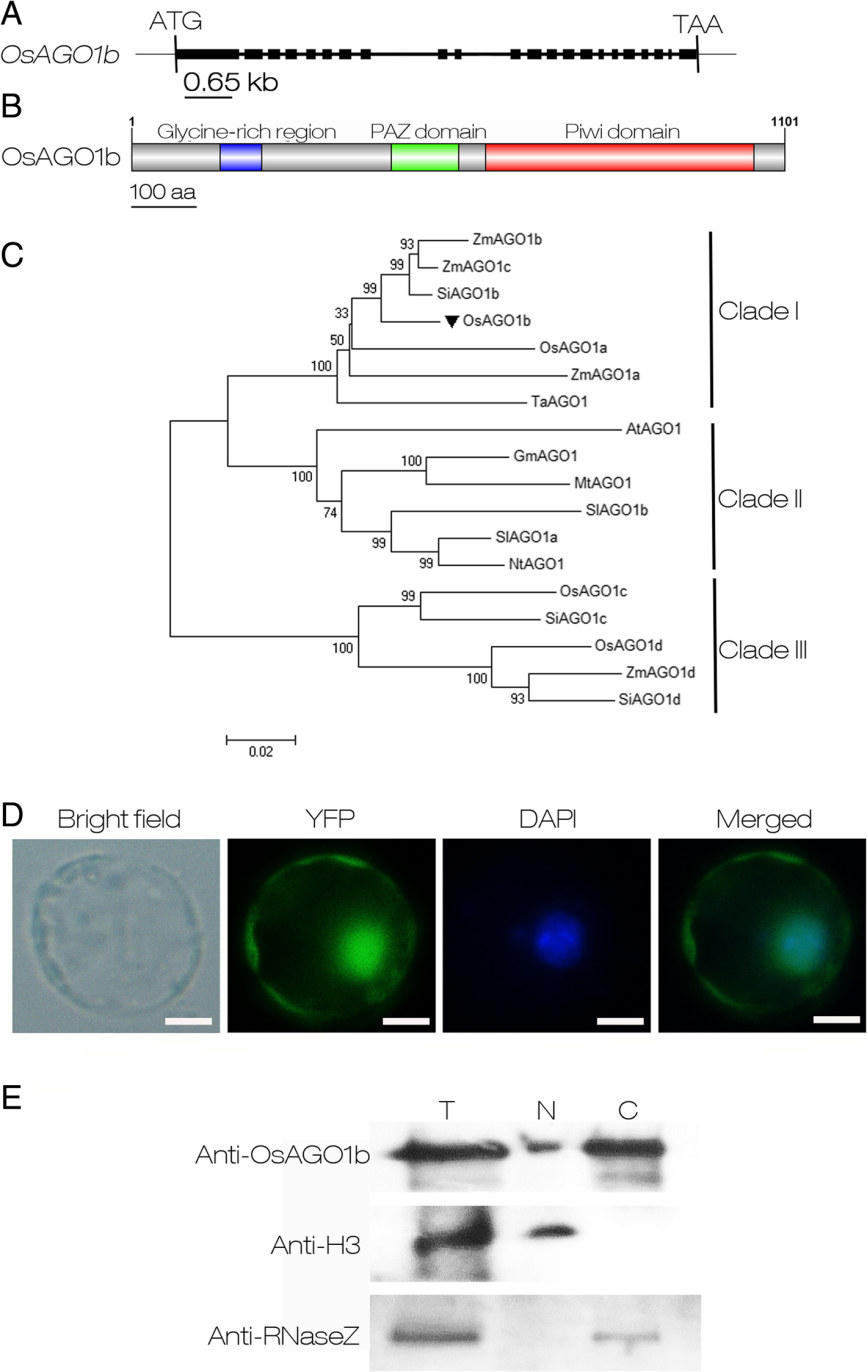
Scheme of the OsAGO1b gene and characterization of the OsAGO1b protein. **a** Genomic organization of the OsAGO1b gene. The closed black boxes indicate exons, and connecting lines inidcate introns. The ATG start codon and TAA stop codon are also indicated. **b** Diagram of the OsAGO1b protein sequence and its main domains. The blue, green and red boxes indicate the Glycine-rice region, PAZ and Piwi domains, respectively. **c** Phylogenic analysis of AGO1 proteins from different plant species. The coding region sequences were aligned using Clustal W, and the evolutionary relationship was analyzed using the neighbor-joining method. Numbers on branches indicate the percentage of replicate trees in which the associated sequences clustered together in the bootstrap test (1000 replicates). The segment under the phylogenic tree is the evolutionary distance, which was computed using the Poisson correction method. The NCBI accesssion numbers for the proteins are presented in Additional file 3: Table S3. **d** Subcelluar localization of the YFP-OsAGO1b fusion protein in leaf sheath protoplasts isolated from 14-day-old seedlings of wild type Zhonghua 11 (ZH11). Scale bars = 10 μm. **e** Western blot analysis of OsAGO1b in nucleoprotein and cytoplasmic proteins isolated from 14-day-old ZH11 seedlings. T, total protein; N, nucleoprotein; C, cytoplasmic protein. Anti-OsAGO1b is the antibody of the OsAGO1b protein. Anti-H3 is the antibody of H3 protein. Anti-RNaseZ is the antibody of the RNaseZ protein

According to the information in NCBI, *OsAGO1b* is located on rice chromosome 4. There are two transcript variants for *OsAGO1b* in rice, i.e. transcript X1 (XM_015780805, 3785 bp) and transcript X2 (XM_015780806, 3825 bp) encoding OsAGO1b isoform X1 (XP_015636291, 1118 aa) and isoform X2 (XP_015636292, 1101 aa) respectively. The amino acids 1–17 of isoform X1 are missing in isoform X2 according to the UniProt record (http://www.uniprot.org/uniprot/Q7XSA2). All the data presented in this study used the *OsAGO1b* transcript variant X2 and the OsAGO1 isoform X2 as in the previous studies (Kapoor et al. 2008; Wu et al. 2009). The annotated genomic sequence of *OsAGO1b* (AL662954.2) contains 22 exons and 21 introns for isoform X2 (Fig. 3a). The intact coding sequence of the *OsAGO1b* transcript variant X2 is 3306 bp, encoding a peptide with a molecular weight of 121.6 kD and a theoretical PI of 9.55 predicted by the ProtParam program (http://web.expasy.org/protparam/). The annotation of the functional domains of OsAGO1b based on a search of the Conserved Domain Database of NCBI (https://www.ncbi.nlm.nih.gov/cdd) indicated that OsAGO1b has a Glycine-rich domain, PAZ domain, and Piwi domain that has significant similarity with RNaseH and possesses the DDH (Asp-Asp-His) catalytic motif required for endonucleolytic cleavage of the target RNA (Fig. 3b; Additional file 8: Figure S5; Rivas et al. 2005). The conserved PAZ and PIWI domains shared by AtAGO1 and three other OsAGO1s suggest that OsAGO1s may recruit small RNAs and possess endonuclease activity. Although the PAZ domain is highly conserved among four OsAGO1s, some polymorphic amino acids exist (Additional file 8: Figure S5), suggesting the small RNAs recruited by each AGO1 might be partially different, as presented for OsAGO1a, OsAGO1b, and OsAGO1c in a previous study (Wu et al. 2009).

Phylogenetic analysis showed the 18 plant AGO1s are classified into three clades. OsAGO1b is closely related to ZmAGO1b, ZmAGO1c, SiAGO1b, and is also closely related to OsAGO1a, ZmAGO1a and TaAGO1; thus, they form Clade I specific to monocot crops. The AGO1s from dicotyledonous plants are clustered together to form Clade II including AtAGO1, GmAGO1, MtAGO1, SlAGO1b, SlAGO1a, and NtAGO1. Clade I is closely related to Clade II, implying OsAGO1b may have similar functions to AtAGO1. OsAGO1c and OsAGO1d belong to Clade III that is also specific to monocotyledons and is more diverged from Clade I and Clade II, suggesting their function might be distinct from that of AtAGO1 (Fig. 3c).

To investigate the subcellular localization of OsAGO1b, the p35S-YFP-OsAGO1b transient expression vector was constructed and transformed into rice leaf sheath protoplasts as described previously (Yang et al. 2014a). The fluorescence signal of the fusion protein was observed in both the nucleus and the cytoplasm (Fig. 3d). To verify the above result, total, nuclear and cytoplasmic proteins were isolated, fractionalized, and used for Western blot analysis. As shown in Fig. 3e, the nuclear-located histone H3 was detected in total and nuclear proteins, and cytoplasm-located RNase Z (Zhou et al. 2014) was detected in total and cytoplasmic proteins. As expected, OsAGO1b was detected in total, nuclear and cytoplasmic proteins. Thus, we propose that OsAGO1b may function in both the nucleus and the cytoplasm to regulate gene expression, similar to AtAGO1 (Bologna et al. 2018; Liu et al. 2018).

### *OsAGO1b* is the Key Regulator for Rice Growth and Development Among Four *OsAGO1s*

It is noteworthy that transgenic plants with increased or decreased expression of the three other rice *AGO1s*, i.e. *OsAGO1a*, *OsAGO1c*, and *OsAGO1d*, did not exhibit dramatic phenotypic alterations, especially in leaves and tiller numbers at the mature stage (Additional file 9: Figure S6). Except the remarkable phenotypic changes of OE-*AGO1b* lines, it is worth noting that the *OsAGO1b* RNAi lines showed no obviously phenotypes in young leaves and mature plant height, however, the seed setting rates were significantly decreased (Additional file 10: Figure S7; Additional file 11: Figure S8A). To investigate the reasons of lower seed setting rates of *OsAGO1b* RNAi lines, the anatomical observation of spikelets was further performed. In contrast to the six erect anthers in one spikelet of ZH11, most anthers of the *OsAGO1b* RNAi spikelet were extremely curved due to the defective development of some pollen sacs accompanied by the reduced pollen fertility, which indicated that anther patterning was disturbed in *OsAGO1b* RNAi lines (Additional file 11: Figure S8B-D). These results suggested that *OsAGO1b* is the key regulator for the normal growth and development of rice among four rice *OsAGO1* genes.

**Fig. 4:**
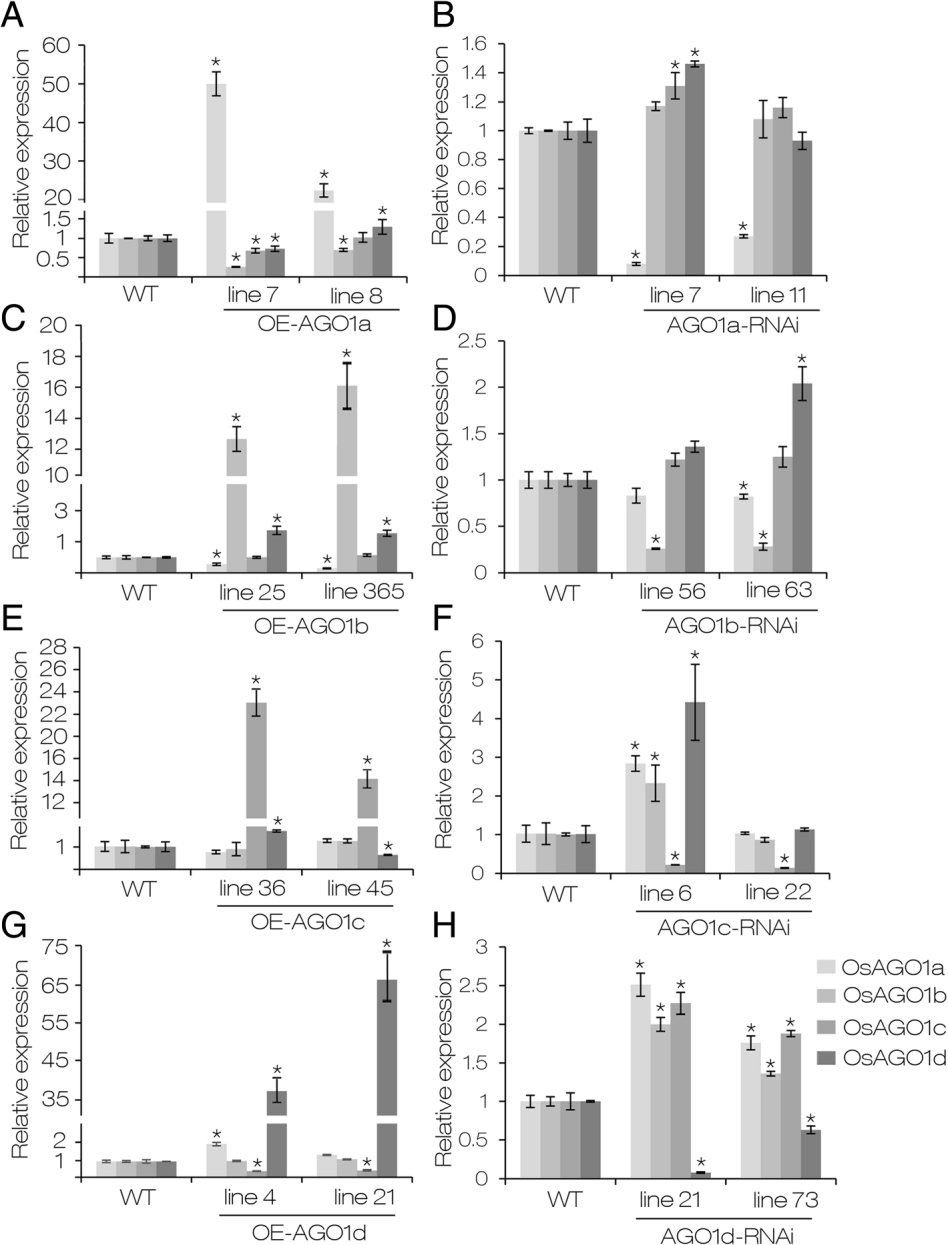
Relative expression of four *AGO1* genes in different overexpression and RNAi lines of *OsAGO1s*. **a**, **c**, **e**, **g** Relative expression of four rice *AGO1* genes in OE-*AGO1a*, OE-*AGO1b*, OE-*AGO1c* and OE-*AGO1d* lines. **b**, **d**, **f**, **h** Relative expression of four rice *AGO1* genes in *AGO1a*-RNAi, *AGO1b*-RNAi, *AGO1c*-RNAi and *AGO1d*-RNAi lines. Flag leaves at the heading stage were used for RNA extraction and qRT-PCR analysis. Means ± SD are given in (**a-h**) (*n* = 3). WT, wild type Zhonghua 11; OE, overexpression. * *P* < 0.05 (one-way ANOVA)

qRT-PCR assay showed that down-regulation/overexpression of one *OsAGO1* gene often caused the increased/decreased expression of another/other *AGO1* member (s) (Fig. 4). For example, *OsAGO1b* overexpression was accompanied by the decreased expression of *OsAGO1a*, and down-regulation of *OsAGO1b* caused an increased expression of *OsAGO1c* and *OsAGO1d* (Fig. 4c and d). Multiple phenotypic changes of the transgenic plants with altered *OsAGO1b* expression suggested that abnormal *OsAGO1b* expression has pleiotropic effects on rice growth and development. It suggested that the complex regulatory loop might exist among rice *AGO1* members.

**Fig. 5:**
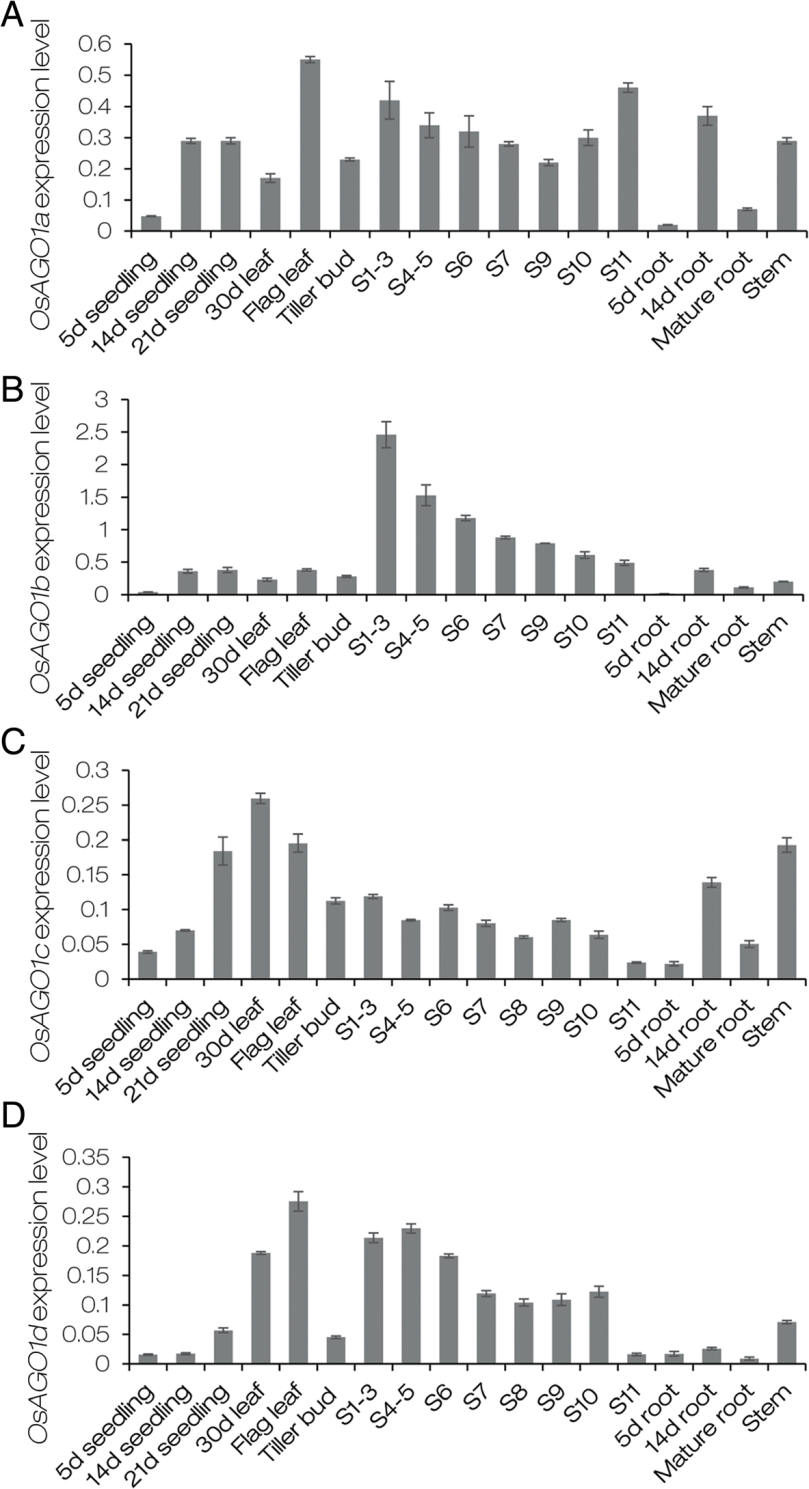
Expression patterns of four rice *AGO1* genes. **a**-**d** Relative expression levels of *OsAGO1a*, *OsAGO1b*, *OsAGO1c* and *OsAGO1d* in various tissues from ZH11 at different developmental stages. 5 d seedlings, 14 d seedlings and 21 d seedlings were harvested from 5-day-old, 14-day-old and 21-day-old seedlings respectively. 30 d leaves indicated the fourth leaves of 30-day-old seedlings. Flag leaves were harvested at the heading stage. S1-S11 indicates spikelets at different developmental stages (Zhang and Wilson 2009). 5 d roots, 14 d roots and mature roots were harvested from 5-day-old, 14-day-old seedlings and heading-stage plants, respectively. Stem tissues were harvested from the third internode of heading-stage plants. The *OsActin1* (XM_015774830) was used as the internal standard to normalize the expression levels of detected genes. Error bars represent standard deviations among replicates (*n* = 3)

To analyze the expression patterns of four *OsAGO1* genes, total RNAs were extracted from various tissues and organs at different developmental stages of ZH11. qRT-PCR assays showed that four *OsAGO1* transcripts were detected in almost all tissues and organs examined; however, different expression patterns were observed (Fig. 5). Notably, *OsAGO1b* showed much higher expression than other *OsAGO1* genes, and the highest expression of *OsAGO1b* was observed in young spikelets at stage 1–3 (S1–3). In 5-day-old seedlings and roots, *OsAGO1b* was expressed at a rather low level and was almost undetectable. In leaves at different developmental stages, *OsAGO1b* was almost unanimously expressed (Fig. 5b). These expression profiles also suggested that the four *OsAGO1s* may share overlapping and distinct functions in rice.

### The Sclerenchymatous Cells are Defective on the Abaxial Side of OE-*AGO1b* Leaves

**Fig. 6:**
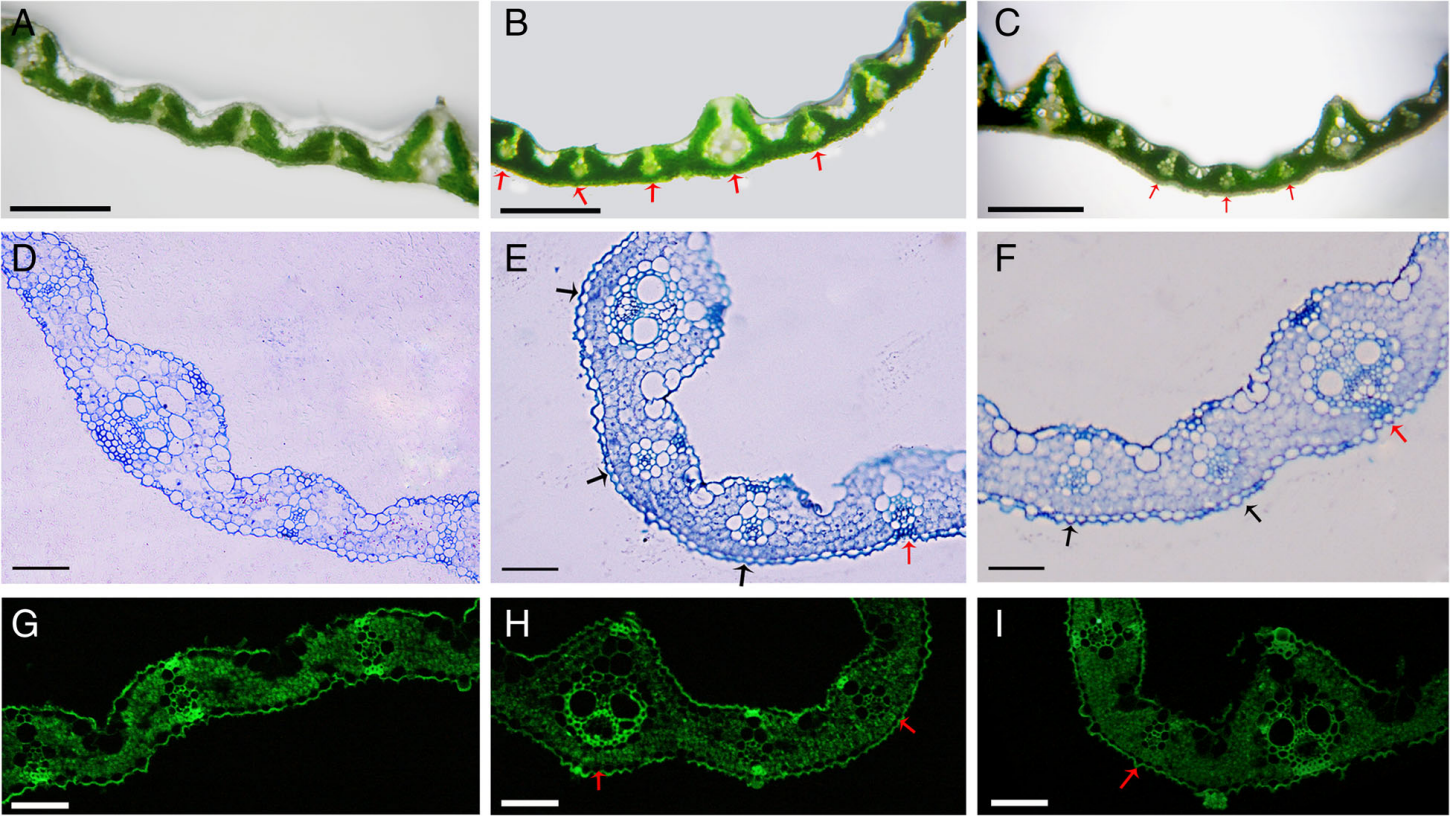
Anatomical observation of wild-type and OE-*AGO1b* leaves. **a**-**c** Freehand cross-sections of 30-day-old seedling leaves (the fourth leaf of the seedling) of wild type ZH11 (**a**), OE-*AGO1b* line 25 (**b**) and line 365 (**c**). Red arrows indicate defective sclerenchymatous cells of the OE-*AGO1b* leaf. Scale bars = 250 μm. **d**-**f** Semi-thin cross-sections of 30-day-old seedling leaves (the fourth leaf of the seedling) of ZH11 (**d**), OE-*AGO1b* line 25 (**e**) and line 365 (**f**). Black arrows and red arrow indicate defective and normal sclerenchymatous cells of the OE-*AGO1b* leaves respectively. Scale bars = 100 μm. **g**-**i** Secondary cell wall autofluorescence of leaves from 30-day-old wild-type (**g**) and OE-*AGO1b* seedlings of line 25 (**h**) and line 365 (**i**). Red arrows indicate defective sclerenchymatous cells with weak autofluorescence on the abaxial side of the OE-*AGO1b* leaf. Scale bars = 50 μm

To determine the cytological cause of adaxially rolled leaves of OE-*AGO1b* plants, the freehand and semi-thin cross-sections of 30-day-old seedlings were observed, and the results revealed that leaf veins had sclerenchymatous cells on the adaxial and abaxial sides of leaves in ZH11, whereas the sclerenchymatous cells on the abaxial side of some small veins were replaced by mesophyll cells in the region where the leaf was curved in OE-*AGO1b* (Fig. 6a-f). Sclerenchymatous cells are generated from mesophyll cells through PCD and are located on the adaxial and abaxial sides of leaf veins. Mesophyll cells undergo secondary cell wall thickening and become dead cells which have thick lignified secondary walls with high amounts of lignin and fiber deposited and provide structural support to a plant (Zhong and Ye 2007). The phenolic compounds of lignin, such as *p*-coumaric acid and ferulic acid, can generate autofluorescence; thus, the autofluorescence intensity can reflect the thickness of the cell wall (Willemse and Den Outer 1988). We then detected the autofluorescence of the leaves from 30-day-old seedling of ZH11 and OE-*AGO1b*. The sclerenchymatous cells of ZH11 leaves showed conspicuous green fluorescence signals on the both sides of the leaf veins. However, no fluorescence signals were detected on the abaxial side of some leaf veins in OE-*AGO1b* (Fig. 6g-i), indicating the abaxial sclerenchymatous cells were developmentally impaired. Our results suggested that OsAGO1b might be involved in leaf adaxial-abaxial polarity development or abaxial side cell differentiation, and it is possible that the defect in sclerenchyma formation in OE-*AGO1b* could be due to more upstream defects such as mis-specification of cellular identity.

### *OsAGO1b* Transcripts do not Show Adaxial-Abaxial Polarity Distribution During Rice Leaf Development

**Fig. 7:**
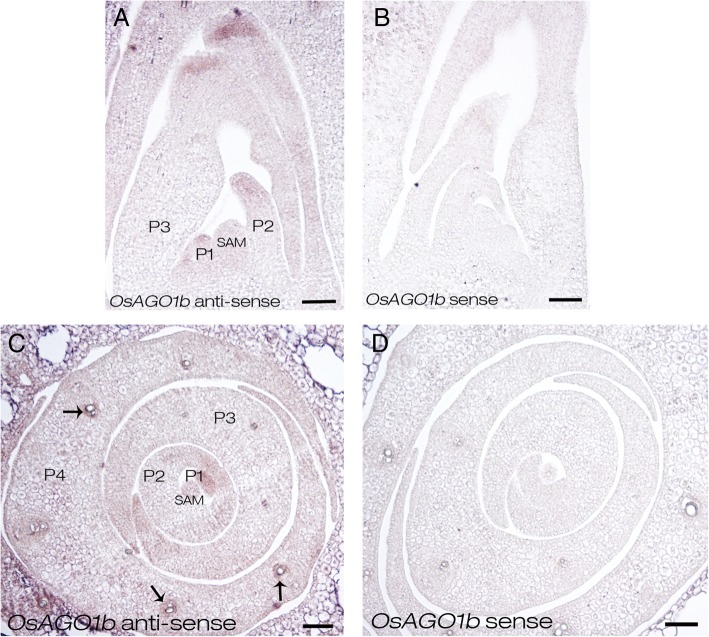
In situ hybridization of *OsAGO1b* in the vegetative shoot apex in ZH11. **a** and **c** In situ hybridization of *OsAGO1b* in ZH11 using anti-sense probe. **b** and **d** In situ hybridization of *OsAGO1b* in ZH11 using sense probe. Shoot base cuttings about 1 cm in length including SAM of 14-day-old seedlings were used. **a**-**b** Longitudinal sections of 14-day-old seedlings. **c**-**d** Cross sections of 14-day-old seedlings. SAM, shoot apical meristem; P1, P1 leaf primordium; P2, P2 leaf primordium; P3, P3 leaf primordium. Black arrows indicate the developing vasculars. Scale bars = 50 μm

Previous studies have shown that the transcripts of some genes involved in leaf polarity development displayed adaxial-abaxial polarity distribution in leaves (Juarez et al. 2004; Fahlgren et al. 2006; Hunter et al. 2006; Zhou et al. 2013). To determine whether *OsAGO1b* mRNAs show polarity distribution, in situ hybridization was performed to detect the expression pattern of *OsAGO1b* in the vegetative shoot apex of ZH11. During leaf development *OsAGO1b* transcripts were visibly accumulating in P1 to P3 primordia without obviously abaxial-adaxial polarity distribution, and it was weakly expressed in SAM. In P4 primordum, *OsAGO1b* mRNAs were detected around vascular bundles (Fig. 7a and c). No visible signal was detected by the sense probe (Fig. 7b and d). These results indicated that the expression of *OsAGO1b* did not show polarity distribution during leaf development, and *OsAGO1b* may be involved in the abaxial side cell differentiation indirectly or directly. Meanwhile, *OsAGO1b* may be participating in the differentiation of vascular tissue.

### SLL1 and SRL2 are Not Directly Involved in the Defective Development of Sclerenchymatous Cells of OE-*AGO1b* Leaves

**Fig. 8:**
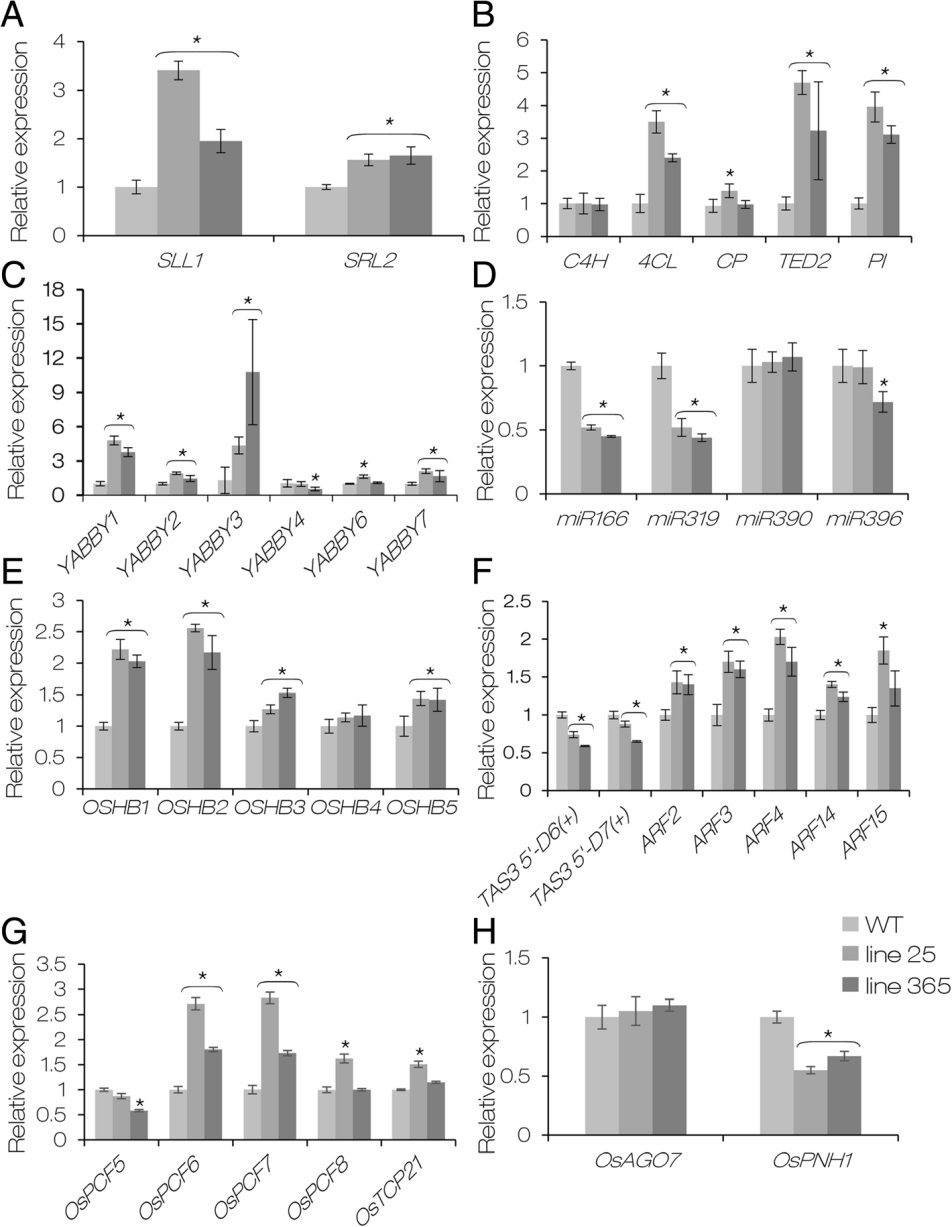
Relative expression of genes and small RNAs related to sclerenchymatous cell development and leaf development. **a** Relative expression of *SLL1* and *SRL2*. **b** Relative expression of tracheary element-PCD related genes. *C4H*, *cinnamate 4-hydroxylase*; *4CL*, *4-coumarate:CoA ligase*; *CP*, *Cys protease*; *TED2*, *tracheary element dehydrogenase 2*; *PI*, *protease inhibitor*. **c** Relative expression of six rice *YABBY* genes. **d** Relative expression of four miRNAs related to leaf development. **e** Relative expression of *OSHB* family genes targeted by miR166. **f** Relative expression of two *TAS3*-tasiRNAs and their targeted *ARF* genes. **g** Relative expression of miR319 targeted genes. **h** Relative expression of *OsAGO7* and *OsPNH1* involved in leaf development. The fourth leaves of 30-day-old seedlings from wild-type Zhonghua 11 (WT) and OE-AGO1b plants were used. The expression level of each gene was normalized to 1 in WT. *OsActin1* (XM_015774830) and the 5S ribosomal RNA gene *rrn5* (NC_011033.1282532..282653, complement) were used as internal standards to normalize the expression levels of genes and small RNAs, respectively. Error bars represent standard deviations among replicates (*n* = 3). * *P* < 0.05 (one-way ANOVA)

SLL1 and SRL2 have been proved to play important roles in regulating abaxial sclerenchymatous cell formation through distinct pathways in rice leaves (Zhang et al. 2009; Liu et al. 2016). The phenotype of the adaxially rolled leaves caused by defective sclerenchymatous cell formation of OE-*AGO1b* was very similar to that observed in the *sll1* and *srl2* mutants, suggesting SLL1 and SRL2 might be involved in the rolled leaf formation of OE-*AGO1b* plants. We then checked the expression levels of *SLL1* and *SRL2* in ZH11 and OE-*AGO1b*. The results showed that the expression levels of *SLL1* and *SRL2* were increased in OE-*AGO1b* compared with those in ZH11 (Fig. 8a), suggesting SLL1 and SRL2 may not directly influence the adaxial sclerenchymatous cell formation in OE-*AGO1b* leaves. Sclerenchymatous cells are transdifferentiated from mesophyll cells in three stages, i.e. dedifferentiation (stage I), conversion of mesophyll cells into tracheary element (TE element) precursor cells (stage II), and TE-specific secondary wall thickening followed by lignification and PCD (stage III) (Fukuda 2000). SLL1 participates in the TE-PCD process by regulating the expression of TE-PCD related genes (Zhang et al. 2009). We then checked the expression of five TE-PCD related genes, i.e. *C4H* (cinnamate 4-hydroxylase), *4CL* (4-coumarate-CoA ligase), *CP* (Cys protease), *TED2* (tracheary element dehydrogenase 2), and *PI* (protease inhibitor) (Zhang et al. 2009). The expression levels of *4CL*, *TED2* and *PI* were significantly increased in OE-*AGO1b*, whereas the expression changes of *C4H* and *CP* were relatively small (Fig. 8b), which suggested that overexpression of *OsAGO1b* might affect the TE-PCD process of abaxial mesophyll cells through regulating the expression of TE-PCD related genes.

In rice, it has been demonstrated that *YABBYs* are not involved in the regulation of leaf polarity formation like in *Arabidopsis*, but participate in cell growth and differentiation, as well as vascular/TE element development of leaves (Liu et al. 2007b; Toriba et al. 2007). Rice contains eight *YABBYs*, named *OsYABBY1* to *OsYABBY7*, and *DROOPING LEAF* (Yamaguchi et al. 2004; Toriba et al. 2007). In the *srl2* mutant, the expression of several *YABBY* genes was decreased significantly (Liu et al. 2016). We then analyzed the expression of *OsYABBY1* to *OsYABBY7*. In contrast to *srl2*, the expression of *OsYABBY5* was almost undetectable in the leaves of 30-day-old seedlings. And the expression levels of *OsYABBY1*, *OsYABBY2*, *OsYABBY3* and *OsYABBY7* were significantly increased in OE-*AGO1b* leaves (Fig. 8c). In general, these results suggested that SLL1 and SRL2 may not directly influence the adaxial sclerenchymatous cell formation in OE-*AGO1b* leaves.

### Overexpression of *OsAGO1b* Affects the Abundance of Small RNAs and their Target Genes Related to Leaf Polarity Development

Some miRNAs have been shown to be involved in the regulation of leaf polarity development, such as miR166 (Reinhart et al. 2002; Williams et al. 2005b), miR319 (Schommer et al. 2008; Yang et al. 2013), miR390 (Douglas et al. 2010) and miR396 (Wang et al. 2011a; Mecchia et al. 2013). To see whether small RNAs may be involved in the development of rolled leaves in OE-*AGO1b* plants, we detected the accumulation changes of four miRNAs, i.e. miRNA166f, miR319b, miR390, and miR396a. The results showed that the accumulation of miR166f and miR319b was significantly reduced, whereas miR390 and miR396a did not show obviously altered accumulation in at least one *OE-AGO1b* line compared with those in ZH11 (Fig. 8d).

*HD-ZIP III* family genes are the targets of miR165/166, and miR165/166 can regulate leaf adaxial-abaxial polarity development by suppressing the expression of *HD-ZIP III* genes on the abaxial side of the leaf (Reinhart et al. 2002; Bowman 2004; Kidner and Martienssen 2004; Byrne 2006). In rice, there are five *HD-ZIP III* genes named *OSHB1* to *OSHB5*, and overexpression of *OSHB1*, *OSHB3*, *OSHB5* respectively caused adaxially rolled leaves with defective sclerenchymatous cells on the abaxial side (Itoh et al. 2008a), which is similar to *OE-AGO1b* plants. We then detected the expression of *OSHB* genes. The results showed that, except for *OSHB4*, the four other genes exhibited significantly increased expression in OE-*AGO1b* leaves (Fig. 8e). Accordingly, we propose that the increased expression of some *OSHB* genes might be the key reason for the adaxially rolled leaves of OE-*AGO1b* plants.

On the other hand, *TAS3-*tasiRNAs are involved in leaf polarity development by suppressing the expression of their target genes, i.e. *ARF2*, *ETT/ARF3*, and *ARF4*, on the abaxial leaf side of *Arabidopsis* (Williams et al. 2005a; Hunter et al. 2006; Husbands et al. 2015). In rice, *TAS3*-tasiRNA 5′-*D6* (+) and *TAS3*-tasiRNA 5′-*D7* (+) are generated from *OsTAS3* loci (Liu et al. 2007a), and there are five orthologous genes of *Arabidopsis ARF2*, *ARF3*, and *ARF4*, i.e. *OsARF2*/*ARF3*-like 2/*OsETTIN2*, *OsARF3*/*ARF3*-like B, *OsARF4*/*OsARF2*/*ARF2*-like, *OsARF14*/*ARF3*-like A, and *OsARF15*/*ARF3*-like 1/*OsETTIN1* (Sato et al. 2001; Williams et al. 2005a; Liu et al. 2007a; Wang et al. 2007). To investigate whether the *TAS3-*tasiRNA-*ARF* pathway is involved in the leaf developmental defects of OE-*AGO1b* plants, the expression levels of two *TAS3-*tasiRNAs and five *OsARF* genes were detected. The results showed that two *TAS3*-tasiRNAs exhibited reduced accumulation, and all five *OsARFs* displayed increased expression in OE-*AGO1b* leaves (Fig. 8f). In plants, miR390 guides the generation of tasiRNAs from *TAS3* transcripts to regulate the expression of *ARF* genes (Xia et al. 2017); however, the accumulation of miR390 did not change in OE-*AGO1b* leaves compared with that in ZH11, implying other factors might exist to regulate *TAS3*-tasiRNA generation.

In rice, miR319 was predicted to target five *TCP* genes, i.e. *OsPCF5*, *OsPCF6*, *OsPCF7*, *OsPCF8* and *OsTCP21* (Wang et al. 2014). In OE-*AGO1b* lines, the expression levels of *OsPCF6*, *OsPCF7*, *OsPCF8* and *OsTCP21* were significantly increased, whereas *OsPCF5* was slightly decreased (Fig. 8g). The changed accumulation of miR319 and its target genes might account for the reduced vein numbers and leaf width of OE-*AGO1b*, as found in a previous study (Wang et al. 2014). Two *AGO* members, *OsAGO7* (Nagasaki et al. 2007; Shi et al. 2007; Itoh et al. 2008b) and *OsPNH1* (Nishimura et al. 2002) are proved to be involved in leaf development in rice. To investigate whether *OsAGO7* and *OsPNH1* are involved in the abnormal leaf development of OE-*AGO1b* plants, we analyzed the expression levels of *OsAGO7* and *OsPNH1*. *OsAGO7* expression was slightly increased in OE-*AGO1b*, implying that *OsAGO7* may not be involved in the phenotypic changes of OE-*AGO1b* leaves; however, *OsPNH1* showed significantly decreased expression in OE-*AGO1b* leaves, suggesting that *OsPNH1* might participate in the altered leaf development in OE-*AGO1b* plants (Fig. 8h).

*OsAGO1b*-RNAi seedlings showed normal leaves similar to that in ZH11 (Additional file 10: Figure S7A and B), we then detected the expression of the *OSHBs* and *OsARFs* in the young leaves of *OsAGO1b*-RNAi lines. And the qRT-PCR results showed that most of the genes showed similar expression levels to ZH11, including *OSHB3*, *OSHB4*, *OSHB5*, *ARF2*, and *ARF5*. And only *OSHB1* showed increased expression in both RNAi lines. The accumulation of *OSHB3* and *OsARF4* were only significantly changed in one RNAi lines (Additional file 12: Figure S9). These results suggested that *OsAGO1b* knockdown may not affect the expression of *OSHBs* and *ARFs* during leaf development. In addition, to see whether *OSHBs* were specifically up-regulated in OE-*AGO1b*, we detected the expression of *OSHBs* in young leaves of *OsAGO1a*, *OsAGO1c* and *OsAGO1d* overexpression lines which showed normal leaf phenotypes (Additional file 13: Figure S10A-C). In contrast to OE-*AGO1b* lines, the expression levels of *OSHBs* did not show the uniformly changing trends in *OsAGO1a*, *OsAGO1c* and *OsAGO1d* overexpression lines (Additional file 13: Figure S10D). For instance, the expression of *OSHB1* and *OSHB2* was significantly decreased and *OSHB3* was obviously increased in OE-*AGO1a* lines; *OSHB1* was significantly increased and *OSHB3* to *OSHB5* were decreased in OE-*AGO1c* lines; *OSHB1* to *OSHB4* were somewhat decreased in OE-*AGO1d*. These results indicated *OSHBs* were specifically up-regulated in rolled leaves of OE-*AGO1b*, distinguished from the overexpression lines of other three *OsAGO1s*.

### Overexpression of *OsAGO1b* does not Affect the Distribution of *OSHB3* and *OsARF4* Transcripts

**Fig. 9:**
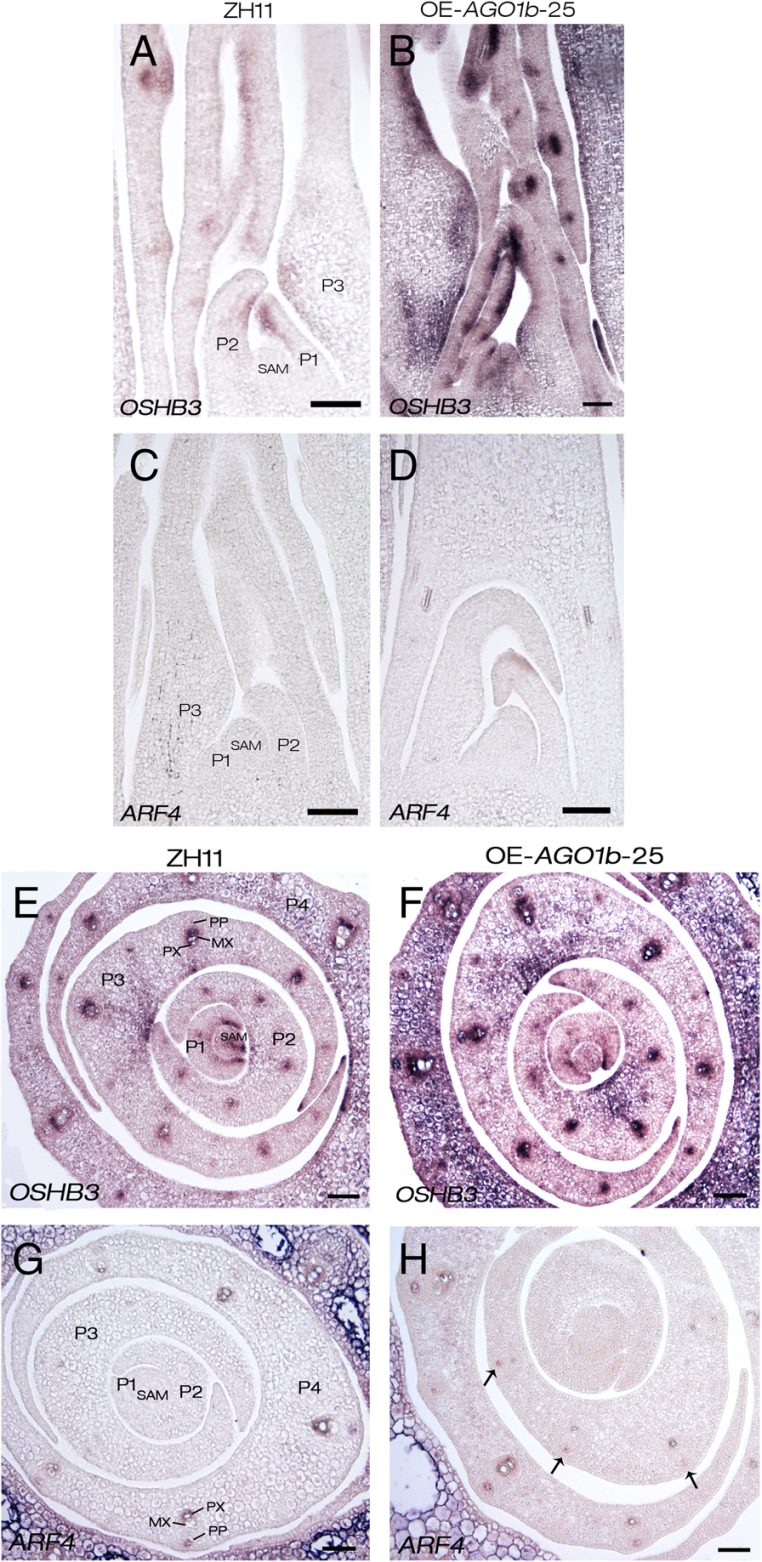
In situ hybridization of *OSHB3* and *OsARF4* in the vegetative shoot apex of ZH11 and OE-*AGO1b*-25. **a** and **b**, **e** and **f** In situ hybridization of *OSHB3* in ZH11 and OE-*AGO1b*-25. **c** and **d**, **g** and **h** In situ hybridization of *ARF4* in ZH11 and OE-*AGO1b*-25. **a**-**d** Longitudinal sections of 14-day-old seedlings. **e**-**h** Cross sections of 14-day-old seedlings. Black arrows in (**h**) showed vascular bundles in P3 primordium of OE-*AGO1b* lines. Shoot base cuttings about 1 cm in length including SAM of 14-day-old seedlings were used. SAM, shoot apical meristem; P1, P1 leaf primordium; P2, P2 leaf primordium; P3, P3 leafprimordium; P4, P4 leaf primordium; MX, metaxylem; PP, protophloem; PX, protoxylem. Scale bars = 50 μm

The expression levels of some genes related to leaf adaxial-abaxial polarity development, such as *OSHBs* and *OsARFs*, were significant increased in OE-*AGO1b* lines as mentioned above. However, whether the distribution of the transcripts of *OSHBs* and *OsARFs* had changed was not clear. To answer this question, we compared the expression patterns of *OSHB3* and *OsARF4* in ZH11 and OE-*AGO1b* lines using in situ hybridization. During the leaf development, the *OSHB3* mRNA was limited to the adaxial sides of P1 to P3 primordia and accumulated in the region where the xylem would develop in ZH11 (Fig. 9a and e; Additional file 14: Figure S11A; Additional file 15: Figure S12A), as previously described (Itoh et al. 2008a). Similarly, in OE-*AGO1b* lines, *OSHB3* mRNA was accumulated in almost the same region compared with ZH11, but the expression was significantly enhanced, consistent with the qRT-PCR results (Fig. 9b and f; Additional file 14: Figure S11B; Additional file 15: Figure S12B). *OsARF4* were weakly expressed in the primary cambiums (protoxylem and protophloem) of vascular bundles from P4 primordium in ZH11 (Fig. 9c and g; Additional file 14: Figure S11C; Additional file 15: Figure S12C). In OE-*AGO1b* lines, the *OsARF4* mRNAs were obviously accumulated on the abaxial sides of leaf primordia as expected, and expressed in the regions of primary cambiums similar to ZH11 (Fig. 9d and h; Additional file 14: Figure S11D; Additional file 15: Figure S12D). However, the *OsARF4* transcripts were mainly first detected in vascular bundles in P3 primordium of OE-*AGO1b* lines (Fig. 9d and h; Additional file 14: Figure S11D; Additional file 15: Figure S12D). In summary, overexpression of *OsAGO1b* may not change the mRNA polarity distributions of *OSHBs* and *OsARFs* during leaf development, but enhanced their expression at the regions of vascular bundles or sclerenchymatous cell differentiation.

## Discussion

### *OsAGO1b* has the Most Important Regulatory Function Among Four Rice *AGO1* Genes

AGO proteins play important roles in plant growth and development through gene regulation mediated by small RNAs, such as miRNAs and siRNAs (Hutvagner and Simard 2008). The *Arabidopsis ago1* mutant displayed a dwarf stem, reduced inflorescence branching, small rosette leaves, and filamentous cauline leaves without adaxial-abaxial differentiation, indicating that AtAGO1 is involved in the regulation of many processes of plant growth and development (Bohmert et al. 1998). Rice contains four *AGO1* members orthologous to *AtAGO1*. A previous work showed simultaneously knockdown four rice *AGO1s* led to pleiotropic developmental defects and the impaired miRNA pathway (Wu et al. 2009). Deep sequencing of small RNAs showed the subset of miRNAs recruited by OsAGO1a, OsAGO1b, and OsAGO1c is different, although most of the miRNAs are almost evenly distributed in the three AGO1 complexes (Wu et al. 2009). In this study, we found that the expression patterns and levels of each *OsAGO1* gene were different in several tissues of different growth stages (Fig. 5), and OsAGO1c and OsAGO1d constitutes a particular clade specific to monocotyledon plants (Fig. 3c). These results suggested although the biological functions of four OsAGO1s may be redundant, it is likely that they have some differences of regulatory functions during rice development. Our study indicated that the expression levels of *OsAGO1b* was higher than other three *AGO1* members and its spatio-temporal expression pattern showed more tissue specific, especially in spikelets of different developmental stages (Fig. 5b). Intriguingly, *OsAGO1b* RNAi lines showed the disturbed anther patterning, decreased pollen fertility and seed setting rates (Additional file 11: Figure S8). A previous study showed that tasiRNA pathway may be involved in regulating adaxial-abaxial polarity in rice stamen development, and the disturbed anther patterning of the *rol* mutant was conferred by a weak mutation in *SHOOTLESS2* encoding an RNA-dependent RNA polymerase (Toriba et al. 2010). The disturbed anther patterning of our *OsAGO1b* RNAi lines was similar to *rol* mutant. Thus, we hypothesize OsAGO1b may be involved in anther patterning and development by regulating tasiRNA pathway, which need to be studied further. In addition, the RNAi lines for each *OsAGO1* gene did not exhibit severe adaxial-abaxial polarity defects on leaves as described in *Arabidopsis ago1* mutants (Bohmert et al. 1998; Vaucheret et al. 2004; Yang et al. 2006), verifying the redundant regulatory functions among four rice *AGO1s*.

Among the overexpression lines of four *OsAGO1s*, only *OsAGO1b* overexpression plants displayed pleiotropic abnormal phenotypes, especially in T_0_ generation lines, such as dark green, severely rolled leaves and decreased root system (Additional file 4: Figure S1A). The phenotypic defects of OE-*AGO1b* seedlings were reminiscent of the phenotypes of the *OsDCL1* RNAi transformants with weak loss of function (Liu et al. 2005) and the RNAi lines with decreased expression of four *OsAGO1* genes simultaneously, and the above *OsDCL1*- and *OsAGO1*- RNAi transformants were both accompanied with the impaired miRNA pathway (Liu et al. 2005; Wu et al. 2009). Moreover, overexpressed miR168-resistant *AGO1* in *Arabidopsis* exhibited impaired miRNA pathway and elevated miRNA targets as well (Vaucheret et al. 2004). Taken together, we propose that ectopic expression of *OsAGO1b* may impair the miRNA pathway and lead to upregulated miRNA targets although other three *OsAGO1s* may have overlapping functions in miRNA pathway. The reduced accumulation of miR166 and miR319 and their up-regulated targets in OE-*AGO1b* leaves supported the speculation above (Fig. 8).

In *Arabidopsis*, different *AGO* members showed functional diversification and redundancy, such as *AGO1* and *AGO10*, which might be dependent or independent on their slicing activity. *Arabidopsis AGO10/PHN/ZLL* exhibits overlapping function with *AGO1* in some developmental processes, such as SAM maintenance and leaf polarity establishment, possibly by competing miR166/165 with AGO1 and repressing miR166/165 (Moussian et al. 1998; Lynn et al. 1999; Fagard et al. 2000; Mallory et al. 2009; Liu et al. 2010; Ji et al. 2011; Zhu et al. 2011; Zhou et al. 2015). Furthermore, protein domain swapping experiments showed that only AGO1 protein with replaced PAZ domain from AGO10 could rescue the developmental defects in *ago1* mutant, but not the AGO1 protein with the replaced AGO10 Piwi domain, suggesting AGO1 and AGO10 could recruit similar miRNAs to regulate SAM and leaf formation, and Piwi domain may contribute to maintain their specific regulatory functions (Mallory et al. 2009). In our study, we found that there were several conserved motifs in the PAZ domains of four OsAGO1s and AtAGO1, however they also had a few diverse amino acid residues between each other, such as three mis-matched residues S-L-S in OsAGO1b (Additional file 8: Figure S5), implying the binding affinity of miRNAs among different OsAGO1s may be different. Similarly, differences in the Piwi domains of each OsAGO1s may cause some different regulatory functions for each OsAGO1 (Additional file 8: Figure S5), similar to AGO1 and AGO10 in *Arabidopsis*, which might partially account for the unique function of OsAGO1b. In addition, the expression of *OSHBs* in four OE-*AGO1s* plants was quite different (Fig. 8e; Additional file 13: Figure S10), implying the distinct regulatory roles of AGO1-miR166-*HD-ZIP III* pathway during leaf morphology formation in rice. Taken together, we propose that OsAGO1b plays the most important regulatory roles in the growth and development of rice among four OsAGO1s, and complex regulatory networks may exist among different AGO proteins in rice.

### Excess of OsAGO1b Induces Elevated miRNA Targets

In plants, miRNAs and siRNAs are important regulators for SAM formation and maintenance, leaf morphogenesis, and leaf polarity formation (Liu et al. 2005; Hunter et al. 2006; Abe et al. 2010; Kidner 2010; Pulido and Laufs 2010; Yang et al. 2018). In this study, we detected the reduced accumulation of miR166, miR319, and two *TAS3*-tasiRNAs, but no changes were found for miR390 and miR396 in OE-*AGO1b* leaves (Fig. 8d). The reduced accumulation of miR166, miR319 and two *TAS3*-tasiRNAs possibly caused the abnormal leaf development in OE-*AGO1b* plants by regulating the expression of their target genes (Fig. 8d, f, and g). The reduced accumulation of miR166 may cause the increased expression of *OSHB* genes, which may result in the adaxially rolled leaves of OE-*AGO1b* plants (Fig. 8e). The excess of OsAGO1b might impair the accumulation of small RNAs by interfering with miRNA stabilization, RISC formation and function, similar to overexpression line of the miR168-resistant *AGO1* in *Arabidopsis* (Vaucheret et al. 2004), suggesting that the excess of AGO1 protein can reduce miRNA accumulation and elevate miRNA targets in both *Arabidopsis* and rice.

A recent study showed that, in addition to binding miRNAs to repress their target genes in cytoplasm, *Arabidopsis* AGO1 can also bind the chromatin of active genes, such as IAA induced genes to promote gene transcription in the nucleus in response to hormones and stresses, suggesting that AGO1 can participate in the regulation of diverse signaling pathways and the corresponding biological processes (Liu et al. 2018). ARFs are transcription factors involved in auxin pathway, and the expression of *OSHBs* could be induced by auxin treatment (Itoh et al. 2008a), thus we speculate excess of OsAGO1b in nucleus may promote the expression of some auxin-induced genes and then stimulate the expression of *ARFs* and *OSHBs*. This may be another reason for the up-regulated expression of *ARFs* and *OSHBs*.

The *Arabidopsis* miR168-resistant *AGO1* plants with a strong increase in *AGO1* accumulation (12.9-fold to 21.7-fold increase) displayed more severe developmental defects, and most of the plants died before flowering (Vaucheret et al. 2004). However, OE-*AGO1b* plants with a 15-fold increase approximately in *OsAGO1b* expression can complete a normal life cycle with almost normal fertility (Fig. 1b). Functional redundancy among different *OsAGO1* members may explain this difference.

### OsAGO1b Might Participate in a New Pathway to Control Sclerenchymatous Cell Development

In OE-*AGO1b* plants, the abaxial sclerenchymatous cells of some small leaf veins were developmentally deficient, and a set of genes and small RNAs involved in leaf polarity development showed changed expression (Fig. 6 and Fig. 8), suggesting OsAGO1b may participate in regulating the abaxial sclerenchymatous cell development by affecting the accumulation of downstream genes dependent or independent of the small RNA pathway. SLL1 and SRL2 are involved in regulating the abaxial sclerenchymatous cell development of leaves through different pathways in rice, and down-regulation of *SLL1* or *SRL2* resulted in the adaxially rolled leaves with defective abaxial sclerenchymatous cells (Zhang et al. 2009; Liu et al. 2016). In OE-*AGO1b* plants, the expression of *SLL1* and *SRL2* were both up-regulated, and *SLL1* overexpression caused twisted and inner rolled leaves with enhanced phloem development on the abaxial side and suppressed sclerenchymatous cell development on the adaxial side of the leaf blade (Zhang et al. 2009), which is different from that of OE-*AGO1b*, suggesting that OsAGO1b might affect the sclerenchymatous cell development in a pathway different from those related to SLL1 and SRL2. In the *sll1* mutant, TE-related genes *CP*, *PI* and *4CL* showed decreased expression, and no obvious expression changes were detected for *TED2* and *C4H* (Zhang et al. 2009). The increased expression of *CP*, *PI* and *4CL* in OE-*AGO1b* plants may be caused by the increased expression of *SLL1*. Notably, *TED2* expression was greatly increased in OE-*AGO1b*, suggesting that OsAGO1b can promote *TED2* expression directly or indirectly (Fig. 8b). *TED2* is the only identified marker gene of stage II of TE-PCD process, and the increased expression of *TED2* suggested that *OsAGO1b* might affect the initiation of stage III events of TE-PCD during sclerenchymatous cell formation (Fukuda 2000). *C4H* and *4CL* are both involved in lignin monomer biosynthesis (Baucher et al. 2003), and the increased *4CL* expression in OE-*AGO1b* suggested that OsAGO1b might affect lignin synthesis.

### The Up-Regulated *OSHB* May Account for Leaf Rolling in *OsAGO1b* Overexpressed Rice Plants

*HD-ZIP III* family genes are involved in SAM maintenance, leaf adaxial cell development, and vascular cell fate determination in *Arabidopsis* (McConnell and Barton 1998; McConnell et al. 2001; Prigge et al. 2005; Ramachandran et al. 2017). Maize *Rld1* encodes a HD-ZIP III protein, and *Rld1* overexpression resulted in upwardly rolled, thread-like leaves (Juarez et al. 2004). Transgenic rice plants with increased expression of *OSHB1* or *OSHB3* exhibited narrow and adaxially rolled, or filamentous leaves and ectopic ligules, and enhanced expression of *OSHB5* led to adaxially rolled leaves with a normal blade-sheath boundary (Itoh et al. 2008a). *OSHB1* overexpression also caused bulliform-like cells on the abaxial side of leaves, and part of the abaxial sclerenchymatous cells exhibited defective development (Itoh et al. 2008a ). *OSHB4/OsHox32* overexpression also produced narrow and upwardly rolled leaves with a deceased number of bulliform cells (Li et al. 2016). The above studies indicate that the suppression of *HD-ZIP III* genes is crucial for the normal development of leaf adaxial-abaxial polarity. In OE-*AGO1b* plants, the expression levels of the five *OSHB* genes were increased overall, and the expression of *OSHB1* and *OSHB2* increased by 2-fold (Fig. 8e), and OE-*AGO1b* showed adaxially rolled leaves with defective abaxial sclerenchymatous cells, similar to the rice plants overexpressing *OSHB* genes (Fig. 6e and f). Notably, the increased expression levels of *OSHBs* in our chosen OE-*AGO1b* lines were much lower than those in the previous study (Itoh et al. 2008a), which might account for the less severe phenotypes of OE-*AGO1b* leaves. One of the possibilities is that some T_0_ plants with severe phenotypes, such as shallot-like rolled leaves, were dead during planting or could not produce seeds (Additional file 4: Figure S1A), indicating the dosage-dependent effect of *OsAGO1b* on the phenotypic changes, which has been proved by the less severe phenotype of the heterozygous OE-*AGO1b* lines (data not shown).

A recent study showed that down-regulation of miR166 generated rice plants with adaxially rolled leaves with abnormal sclerenchymatous cells, accompanied by the increased expression of five *OSHB* genes, and *OSHB4* was proved to be the major target of miR166; *OSHB4* overexpression caused leaf rolling similar to miR166 down-regulated plants, indicating a correlation between the miR166-*OSHB* regulatory pathway and leaf adaxial-abaxial polarity development in rice, similar to *Arabidopsis* (Zhang et al. 2018). In this study, *OsAGO1b* overexpression also resulted in the increased accumulation of *OSHBs* and the decreased accumulation of miR166, suggesting an *OsAGO1b*-miR166-*HD-ZIP III* regulatory pathway in leaf adaxial-abaxial patterning in rice. How the regulatory pathways related to SLL, SRL2, and OsAGO1b respectively coordinate and interact in the regulation of sclerenchymatous cell development needs further study. In addition, *OsHox32/OSHB4* overexpression caused significantly decreased or increased expression of different *YABBY* genes, suggesting that *OSHB4* might participate in leaf development by regulating *YABBY* genes (Li et al. 2016). In OE-*AGO1b* plants, the expression of some *YABBY* genes was also changed; however, *OSHB4* showed a very similar expression to that in the wild type, suggesting the regulation of *YABBY* genes is complicated (Fig. 8c). ATHB4 and HAT3, two HD-ZIP II transcription factors, were also proved to control leaf adaxial/abaxial patterning in *Arabidopsis* (Bou-Torrent et al. 2012; Turchi et al. 2013). And a further study showed that HD-ZIP III and HD-ZIP II protein can act together to directly repress the expression of MIR165/166 genes, indicating a complex regulation circuit between these regulators (Merelo et al. 2016). From our in situ hybridization results, *OsAGO1b*, *OSHB3* and *OsARF4* were all expressed in the regions of vascular tissue formation in this study. Considering that *OSHB3* and *OsARF4* are both auxin inducing genes, our results suggest that *OsAGO1b* may afftect the sclerenchymatous cell formation of the leaf abaxial side through the auxin pathway.

## Conclusions

In summary, our work demonstrated that overexpression of *OsAGO1b* resulted in narrow and adaxially rolled leaves with reduced leaf vein number and defective sclerenchymatous cells, accompanying with the reduced small RNA levels and increased expression of their target genes. This study provided a new perspective to the understanding of the molecular mechanisms governing the sclerenchymatous cell development in rice. We analyzed and compared the protein motifs, expression patterns, transgenic plant phenotypes and related genes expression of four rice *AGO1s*, and our results showed that *OsAGO1b* is the main regulator in rice organ development among four rice *AGO1s*.

## Additional files

Additional file 1: **Table S1.** Primers used in this study. (DOCX 27 kb)

Additional file 2: **Table S2.** Information of the construction of overexpression and RNAi vectors for rice four *AGO1* genes. (DOCX 15 kb)

Additional file 3: **Table S3.** Information of plant AGO1 proteins used for phylogenetic analysis. (DOCX 12 kb)

Additional file 4: **Figure S1.** Characterization of T_0_ transgenic plants of the OE-*AGO1b* lines. (A) Regenerated seedlings for the control empty vector pCAMBIA1380 (left) and *OsAGO1b*-overexpression construct (right). Scale bar = 1 cm. (B) Detection of T-DNA insertion numbers of *OsAGO1b*-overexpression lines using Southern blot analysis. Transgenic lines 25, 40, 365 containing a single copy T-DNA were chosen for further study. OE-*AGO1b*, *OsAGO1b*-overexpression line. (TIF 5135 kb)

Additional file 5: **Figure S2.** Young leaf development of the OE-*AGO1b* line and ZH11. (A-D) indicates 7-day-old, 14-day-old, 21-day-old, and 28-day-old seedlings after germination. Scale bars = 2 cm for (A) and (B). Scale bars = 10 cm for (C) and (D). The *OsAGO1b*-overexpression line displayed adaxially rolled leaves from the fourth leaf stage. OE-*AGO1b*, *OsAGO1b*-overexpression line; ZH11, wild type Zhonghua 11. (TIF 9073 kb)

Additional file 6: **Figure S3.** Agronomic traits of ZH11 and OE-*AGO1b* lines. (A-F) Tiller number, inflorescence branch number, plant height, inflorescence length, grain number per panicle, and seed setting percentage of ZH11 (WT) and OE-*AGO1b* (line 25, line 40, line 365), respectively. Results are shown as the mean ± SD in (A)-(F) (*n* = 10). ** *P* < 0.01 (one-way ANOVA). OE-*AGO1b*, *OsAGO1b*-overexpression line; ZH11, wild type Zhonghua 11. (TIF 1467 kb)

Additional file 7: **Figure S4.** Phenotypes of OE-*AGO1b* lines for different rice varieties. (A-D) Wild type and OE-*AGO1b* plants at the booting stage or heading stage for Nipponbare, Songgeng, Huanghuazhan and Annong. All the plants were geminated and planted at the same time under the same conditions. Scale bars = 15 cm. (E-H) Relative expression of *OsAGO1b* in OE-*AGO1b* plants of Nipponbare (Nip), Songgeng (Sg), Huanghuazhan (HHZ) and Annong (AN) backgrounds. Flag leaves at the booting stage were used for RNA extraction and qRT-PCR analysis. Means ± SD are presented in (E-H) (*n* = 3). * *P* < 0.05, ** *P* < 0.01 (one-way ANOVA). OE-*AGO1b*, *OsAGO1b*-overexpression; WT, wild type. (TIF 8027 kb)

Additional file 8: **Figure S5.** Amino acid sequences of the PAZ and Piwi domains of OsAGO1s and AtAGO1. Alignment of amino acid sequences of the PAZ domain (A) the Piwi domains (B) of OsAGO1s and AtAGO1. “*” indicated the polymorphic amino acid residues specific to OsAGO1b. (TIF 3570 kb)

Additional file 9: **Figure S6.** Phenotypes of four rice *AGO1*-overexpression and RNAi transgenic plants. (A, C, E, G) Morphologies of lines overexpressing *OsAGO1a*, *OsAGO1b*, *OsAGO1c* and *OsAGO1d* at the mature stage. (B, D, F, H) Morphologies of RNAi lines for *OsAGO1a*, *OsAGO1b*, *OsAGO1c* and *OsAGO1d* at the mature stage. Scale bars = 15 cm. WT, wild type Zhonghua 11; OE, overexpression. (TIF 23721 kb)

Additional file 10: **Figure S7.** Phenotypes and agronomic traits of ZH11 and *OsAGO1b* RNAi lines. (A) Phenotypes of 30-day-old seedlings of ZH11 and *OsAGO1b* RNAi lines, scale bar = 10 cm. (B) The leaves of 30-day-old seedlings of ZH11 and *OsAGO1b* RNAi lines, scale bar = 3 cm. (C and D) Plant heights and seed setting rates of ZH11 (WT) and *OsAGO1b* RNAi lines. Results are shown as the mean ± SD in (C)-(D) (*n* = 10). * *P* < 0.05, ** *P* < 0.01 (one-way ANOVA). (TIF 5956 kb)

Additional file 11: **Figure S8.** Phenotypes of spikelets and anthers of ZH11 and *OsAGO1b* RNAi lines. (A) The panicles of ripeness stage, scale bar = 4 cm. (B) Dissection of mature spikelets, the white arrows indicated the curly anthers, scale bar = 1 mm. (C) I_2_-KI staining of pollens. (D) Phenotypes of anthers, the white arrows indicated the aberrant anther sacs, scale bar = 0.5 mm. (TIF 14943 kb)

Additional file 12: **Figure S9.** Relative expression of *OSHB* and *OsARF* genes in WT and *OsAGO1b* RNAi lines. The fourth leaves of 30-day-old seedlings from wild-type Zhonghua 11 (WT) and *OsAGO1b* RNAi line 56 and line 63 were used. The expression level of each gene was normalized to 1 in WT. *OsActin1* (XM_015774830) was used as an internal standard to normalize the expression levels of detected genes. Error bars represent standard deviations among replicates (*n* = 3). * *P* < 0.05, ** *P* < 0.01 (one-way ANOVA). (TIF 1542 kb)

Additional file 13: **Figure S10.** The phenotypes of *OsAGO1a*, *OsAGO1c* and *OsAGO1d* overexpression lines and their expression levels of *OSHBs*. (A-C) Phenotypes of 30-day-old seedlings of ZH11 and *OsAGO1a*, *OsAGO1c* and *OsAGO1d* overexpression lines, scale bars = 10 cm. (D) Relative expression of *OSHBs*. The fourth leaves of 30-day-old seedlings from wild-type Zhonghua 11 (ZH11) and each transgenic line were used for RNA extration. The expression level of each gene was normalized to 1 in WT. *OsActin1* (XM_015774830) was used as an internal standard to normalize the expression levels of detected genes. OE-AGO1 indicated AGO1 overexprssion lines. Error bars represent standard deviations among replicates (*n* = 3). * *P* < 0.05 (one-way ANOVA). (TIF 7387 kb)

Additional file 14: **Figure S11.** In situ hybridization of *OSHB3* and *OsARF4* in the vegetative shoot apex of ZH11 and OE-*AGO1b*-365 (longitudinal sections). (A and C) In situ hybridization of *OSHB3* and *OsARF4* in ZH11 using sense probe. (B and D) In situ hybridization of *OSHB3* and *OsARF4* in OE-*AGO1b*-365 using anti-sense probe. Shoot base cuttings about 1 cm in length including SAM of 14-day-old seedlings were used. Scale bars = 50 μm. (TIF 12330 kb)

Additional file 15: **Figure S12.** In situ hybridization of *OSHB3* and *OsARF4* in the vegetative shoot apex of ZH11 and OE-*AGO1b*-365 (cross sections). (A and C) In situ hybridization of *OSHB3* and *OsARF4* in ZH11 using sense probe. (B and D) In situ hybridization of *OSHB3* and *OsARF4* in OE-*AGO1b*-365 using anti-sense probe. Shoot base cuttings about 1 cm in length including SAM of 14-day-old seedlings were used. Black arrows indicate the developing vasculars. Scale bars = 50 μm. (TIF 16952 kb)

## Data Availability

All data supporting the conclusions of this article are provided with the article and its supplementary information files.

## Abbreviations

*4CL*: *4-coumarate-CoA ligase*
AGO: ARGONAUTE
AN: Annong
*ARF3*/*ETT*: *AUXIN RESPONSE FACTOR 3*/*ETTIN*
*C4H*: *cinnamate 4-hydroxylase*
CaMV: cauliflower mosaic virus
*CP*: *Cys protease*
CTAB: hexadecy ltrimethyl ammonium bromide
*DCL1*: *Dicer-like1*
*HD-ZIP III*/*OSHB*: *Class III homeodomain-leucine zipper*
*HEN1*: *HUA ENHANCER 1*
HHZ: Huanghuazhan
*Hyg*: *Hygromycin phosphotransferase*
*HYL1*: *HYPONASTIC LEAVES 1*
*KAN*: *KANADI*
LEI: Leaf erect index
Lfw: Leaf greatest width of flat state
Lnl: Leaf length of natural state
Lnw: Leaf greatest width of natural state
LRI: Leaf rolling index
Lsl: Leaf length of straighten state
*MEL1*: *MEIOSIS ARRESTED AT LEPTOTENE1*
miRNA: microRNA
Nip: Nipponbare
*NOS*: *nopaline synthase*
OE-*AGO1*: *OsAGO1b*-overexpression
PAGE: Polyacrylamide gel electrophoresis
PCD: Programmed cell death
phasiRNA: Phased small interfering RNA
*PHB*: *PHABULOSA*
*PHV*: *PHAVOLUTA*
*PI*: *Protease inhibitor*
*PNH/ZLL*: *PINHEAD*/*ZWILLE*
pSK: pBluescript II SK (+)
qRT-PCR: Quantitative reverse transcription-PCR
*REV*: *REVOLUTE*
RISC: RNA-induced silencing complex
SAM: Shoot apical meristem
SDS: Sodium dodecyl sulfate
Sg: Songgeng
*SHL4*: *SHOOTLESS 4*
*SHO2*: *SHOOT ORGANIZATION2*
*SLL1*: *SHALLOT-LIKE 1*
*SRL1*: *SEMI-ROLLED LEAF1*
*SRL2*: *Semi-Rolled Leaf 2*
tasiRNA: Trans-acting small interference RNA
TCP/PCF: Teosinte branched1/Cincinnata/proliferating cell factor
TE: Tracheary element
*TED2*: *tracheary element dehydrogenase 2*
*Ubi*: *Ubiqutin*
WT: Wild type
*YFP*: *Yellow florescence protein*
ZH11: Zhonghua11

## References

[CR1] Abe M, Yoshikawa T, Nosaka M, Sakakibara H, Sato Y, Nagato Y, Itoh J (2010). WAVY LEAF1, an ortholog of Arabidopsis *HEN1*, regulates shoot development by maintaining microRNA and trans-acting small interfering RNA accumulation in rice. Plant Physiol.

[CR2] Baucher M, Halpin C, Petit-Conil M, Boerjan W (2003). Lignin: genetic engineering and impact on pulping. Crit Rev Biochem Mol Biol.

[CR3] Baulcombe D (2004). RNA silencing in plants. Nature.

[CR4] Bohmert K, Camus I, Bellini C, Bouchez D, Caboche M, Benning C (1998). AGO1 defines a novel locus of *Arabidopsis* controlling leaf development. EMBO J.

[CR5] Bologna NG, Iselin R, Abriata LA, Sarazin A, Pumplin N, Jay F, Grentzinger T, Dal Peraro M, Voinnet O (2018). Nucleo-cytosolic shuttling of ARGONAUTE1 prompts a revised model of the plant microRNA pathway. Mol Cell.

[CR6] Bou-Torrent J, Salla-Martret M, Brandt R, Musielak T, Palauqui JC, Martínez-García JF, Wenkel S (2012). ATHB4 and HAT3, two class II HD-ZIP transcription factors, control leaf development in *Arabidopsis*. Plant Signal Behav.

[CR7] Bowman JL (2004). Class III HD-zip gene regulation, the golden fleece of ARGONAUTE activity?. Bioessays.

[CR8] Bowman JL, Eshed Y, Baum SF (2002). Establishment of polarity in angiosperm lateral organs. Trends Genet.

[CR9] Braybrook SA, Kuhlemeier C (2010). How a plant builds leaves. Plant Cell.

[CR10] Byrne ME (2006). Shoot meristem function and leaf polarity: the role of class III HD-ZIP genes. PLoS Genet.

[CR11] Chen J, Yu J, Ge L, Wang H, Berbel A, Liu Y, Chen Y, Li G, Tadege M, Wen J, Cosson V, Mysore KS, Ratet P, Madueño F, Bai G, Chen R (2010). Control of dissected leaf morphology by a Cys (2)his(2) zinc finger transcription factor in the model legume *Medicago truncatula*. Proc Natl Acad Sci U S A.

[CR12] Chen Q, Xie Q, Gao J, Wang W, Sun B, Liu B, Zhu H, Peng H, Zhao H, Liu C, Wang J, Zhang J, Zhang G, Zhang Z (2015). Characterization of rolled and erect leaf 1 in regulating leave morphology in rice. J Exp Bot.

[CR13] Douglas RN, Wiley D, Sarkar A, Springer N, Timmermans MC, Scanlon MJ (2010). *ragged seedling2* encodes an ARGONAUTE7-like protein required for mediolateral expansion, but not dorsiventrality of maize leaves. Plant Cell.

[CR14] Du P, Wu J, Zhang J, Zhao S, Zheng H, Gao G, Wei L, Li Y (2011). Viral infection induces expression of novel phased microRNAs from conserved cellular microRNA precursors. PLoS Pathog.

[CR15] Fagard M, Boutet S, Morel JB, Bellini C, Vaucheret H (2000). AGO1, QDE-2, and RDE-1 are related proteins required for post-transcriptional gene silencing in plants, quelling in fungi, and RNA interference in animals. Proc Natl Acad Sci U S A.

[CR16] Fahlgren N, Montgomery TA, Howell MD, Allen E, Dvorak SK, Alexander AL, Carrington JC (2006). Regulation of *AUXIN RESPONSE FACTOR3* by *TAS3* ta-siRNA affects developmental timing and patterning in *Arabidopsis*. Curr Biol.

[CR17] Fang X, Qi Y (2016). RNAi in plants: an Argonaute-centered view. Plant Cell.

[CR18] Felsenstein J (1985). Confidence limits on phylogenies: an approach using the bootstrap. Evolution.

[CR19] Fukuda H (2000). Programmed cell death of tracheary elements as a paradigm in plants. Plant Mol Biol.

[CR20] Garcia D, Collier SA, Byrne ME, Martienssen RA (2006). Specification of leaf polarity in *Arabidopsis* via the *trans*-acting siRNA pathway. Curr Biol.

[CR21] Hiei Y, Ohta S, Komari T, Kumashiro T (1994). Efficient transformation of rice (*Oryza sativa* L.) mediated by *Agrobacterium* and sequence analysis of the boundaries of the T-DNA. Plant J.

[CR22] Horton P (2000). Prospects for crop improvement through the genetic manipulation of photosynthesis: morphological and biochemical aspects of light capture. J Exp Bot.

[CR23] Hu J, Zhu L, Zeng D, Gao Z, Guo L, Fang Y, Zhang G, Dong G, Yan M, Liu J, Qian Q (2010). Identification and characterization of *NARROW AND ROLLED LEAF 1*, a novel gene regulating leaf morphology and plant architecture in rice. Plant Mol Biol.

[CR24] Hu X, Liu Y-G (2006). The construction of RNAi vectors and the use for gene silencing in rice. Mol Plant Breeding.

[CR25] Hunter C, Willmann MR, Wu G, Yoshikawa M, de la Luz Gutierrez-Nava M, Poethig SR (2006). Trans-acting siRNA-mediated repression of ETTIN and ARF4 regulates heteroblasty in *Arabidopsis*. Development.

[CR26] Husbands AY, Benkovics AH, Nogueira FT, Lodha M, Timmermans MC (2015). The ASYMMETRIC LEAVES complex employs multiple modes of regulation to affect adaxial-abaxial patterning and leaf complexity. Plant Cell.

[CR27] Hutvagner G, Simard MJ (2008). Argonaute proteins: key players in RNA silencing. Nat Rev Mol Cell Bio.

[CR28] Itoh J, Hibara K, Sato Y, Nagato Y (2008). Developmental role and auxin responsiveness of class III homeodomain leucine zipper gene family members in rice. Plant Physiol.

[CR29] Itoh J, Sato Y, Nagato Y (2008). The *SHOOT ORGANIZATION2* gene coordinates leaf domain development along the central-marginal axis in rice. Plant Cell Physiol.

[CR30] Ji L, Liu X, Yan J, Wang W, Yumul RE, Kim YJ, Dinh TT, Liu J, Cui X, Zheng B, Agarwal M, Liu C, Cao X, Tang G, Chen X (2011). ARGONAUTE10 and ARGONAUTE1 regulate the termination of floral stem cells through two microRNA in *Arabidopsis*. PLoS Genet.

[CR31] Juarez MT, Twigg RW, Timmermans MC (2004). Specification of adaxial cell fate during maize leaf development. Development.

[CR32] Kapoor M, Arora R, Lama T, Nijhawan A, Khurana JP, Tyagi AK, Kapoor S (2008). Genome-wide identification, organization and phylogenetic analysis of dicer-like, Argonaute and RNA-dependent RNA polymerase gene families and their expression analysis during reproductive development and stress in rice. BMC Genomics.

[CR33] Kerstetter RA, Bollman K, Taylor RA, Bomblies K, Poethig RS (2001). KANADI regulates organ polarity in *Arabidopsis*. Nature.

[CR34] Kidner CA (2010). The many roles of small RNAs in leaf development. J Genet Genomics.

[CR35] Kidner CA, Martienssen RA (2004). Spatially restricted microRNA directs leaf polarity through ARGONAUTE1. Nature.

[CR36] Kidner CA, Timmermans MC (2007). Mixing and matching pathways in leaf polarity. Curr Opin Plant Biol.

[CR37] Komiya R, Ohyanagi H, Niihama M, Watanabe T, Nakano M, Kurata N, Nonomura K (2014). Rice germline-specific Argonaute MEL1 protein binds to phasiRNAs generated from more than 700 lincRNAs. Plant J.

[CR38] Kumar S, Stecher G, Tamura K (2016). MEGA7: molecular evolutionary genetics analysis version 7.0 for bigger datasets. Mol Biol Evol.

[CR39] Li J, Jiang D, Zhou H, Li F, Yang J, Hong L, Fu X, Li Z, Liu Z, Li J, Zhuang C (2011). Expression of RNA interference/antisense transgenes by the cognate promoters of target genes is a better gene-silencing strategy to study gene functions in rice. PLoS One.

[CR40] Li M, Xiong G, Li R, Cui J, Tang D, Zhang B, Pauly M, Cheng Z, Zhou Y (2009). Rice cellulose synthase-like D4 is essential for normal cell-wall biosynthesis and plant growth. Plant J.

[CR41] Li YY, Shen A, Xiong W, Sun QL, Luo Q, Song T, Li ZL, Luan WJ (2016). Overexpression of *OsHox32* results in pleiotropic effects on plant type architecture and leaf development in rice. Rice.

[CR42] Liang J, Guo S, Sun B, Liu Q, Chen X, Peng H, Zhang Z, Xie Q (2018). Constitutive expression of *REL1* confers the rice response to drought stress and abscisic acid. Rice (N Y).

[CR43] Liu B, Chen Z, Song X, Liu C, Cui X, Zhao X, Fang J, Xu W, Zhang H, Wang X, Chu C, Deng X, Xue Y, Cao X (2007). Oryza sativa dicer-like4 reveals a key role for small interfering RNA silencing in plant development. Plant Cell.

[CR44] Liu B, Li P, Li X, Liu C, Cao S, Chu C (2005). Loss of function of *OsDCL1* affects microRNA accumulation and causes developmental defects in rice. Plant Physiol.

[CR45] Liu C, Xin Y, Xu L, Cai Z, Xue Y, Liu Y, Xie D, Liu Y, Qi Y (2018). *Arabidopsis* ARGONAUTE1 binds chromatin to promote gene transcription in response to hormones and stresses. Dev Cell.

[CR46] Liu H, Nonomura KI (2016). A wide reprogramming of histone H3 modifications during male meiosis I in rice is dependent on the Argonaute protein MEL1. J Cell Sci.

[CR47] Liu H, Xu Y, Xu Z, Chong K (2007). A rice *YABBY* gene, *OsYABBY4*, preferentially expresses in developing vascular tissue. Dev Genes Evol.

[CR48] Liu Q, Yao X, Pi L, Wang H, Cui X, Huang H (2010). The *ARGONAUTE10* gene modulates shoot apical meristem maintenance and establishment of leaf polarity by repressing miR165/166 in Arabidopsis. Plant J.

[CR49] Liu X, Li M, Liu K, Tang D, Sun M, Li Y, Shen Y, Du G, Cheng Z (2016). *Semi-Rolled Leaf2* modulates rice leaf rolling by regulating abaxial side cell differentiation. J Exp Bot.

[CR50] Livak KJ, Schmittgen TD (2001). Analysis of relative gene expression data using real-time quantitative PCR and the 2^−ΔΔ*C*^_T_ method. Methods.

[CR51] Lynn K, Fernandez A, Aida M, Sedbrook J, Tasaka M, Masson P, Barton MK (1999). The *PINHEAD/ZWILLE* gene acts pleiotropically in *Arabidopsis* development and has overlapping functions with the *ARGONAUTE1* gene. Development.

[CR52] Mallory A, Hinze A, Tucker MR, Bouché N, Gasciolli V, Elmayan T, Lauressergues D, Jauvion V, Vaucheret H, Laux T (2009). Redundant and specific roles of the ARGONAUTE proteins AGO1 and ZLL in development and small RNA-directed gene silencing. PLoS Genet.

[CR53] Mallory A, Vaucheret H (2010). Form, function, and regulation of ARGONAUTE proteins. Plant Cell.

[CR54] McConnell JR, Barton MK (1998). Leaf polarity and meristem formation in *Arabidopsis*. Development.

[CR55] McConnell JR, Emery J, Eshed Y, Bao N, Bowman J, Barton MK (2001). Role of *PHABULOSA* and *PHAVOLUTA* in determining radial patterning in shoots. Nature.

[CR56] Mecchia MA, Debernardi JM, Rodriguez RE, Schommer C, Palatnik JF (2013). MicroRNA miR396 and RDR6 synergistically regulate leaf development. Mech Dev.

[CR57] Merelo P, Ram H, Pia Caggiano M, Ohno C, Ott F, Straub D, Graeff M, Cho SK, Yang SW, Wenkel S, Heisler MG (2016). Regulation of MIR165/166 by class II and class III homeodomain leucine zipper proteins establishes leaf polarity. Proc Natl Acad Sci U S A.

[CR58] Moon J, Hake S (2011). How a leaf gets its shape. Curr Opin Plant Biol.

[CR59] Moussian B, Schoof H, Haecker A, Jürgens G, Laux T (1998). Role of the *ZWILLE* gene in the regulation of central shoot meristem cell fate during *Arabidopsis* embryogenesis. EMBO J.

[CR60] Nagasaki H, Itoh J, Hayashi K, Hibara K, Satoh-Nagasawa N, Nosaka M, MukouhataM AM, Kitano H, Matsuoka M, Nagato Y, Sato Y (2007). The small interfering RNA production pathway is required for shoot meristem initiation in rice. Proc Natl Acad Sci U S A.

[CR61] Nishimura A, Ito M, Kamiya N, Sato Y, Matsuoka M (2002). *OsPNH1* regulates leaf development and maintenance of the shoot apical meristem in rice. Plant J.

[CR62] Nonomura K, Morohoshi A, Nakano M, Eiguchi M, Miyao A, Hirochika H (2007). A germ cell specific gene of the ARGONAUTE family is essential for the progression of premeiotic mitosis and meiosis during sporogenesis in rice. Plant Cell.

[CR63] Otsuga D, DeGuzman B, Prigge MJ, Drews GN, Clark SE (2008). *REVOLUTA* regulates meristem initiation at lateral positions. Plant J.

[CR64] Prigge MJ, Otsuga D, Alonso JM, Ecker JR, Drews GN, Clark SE (2005). Class III homeodomain-leucine zipper gene family members have overlapping, antagonistic, and distinct roles in Arabidopsis development. Plant Cell.

[CR65] Pulido A, Laufs P (2010). Co-ordination of developmental processes by small RNAs during leaf development. J Exp Bot.

[CR66] Ramachandran P, Carlsbecker A, Etchells JP (2017). Class III HD-ZIPs govern vascular cell fate: an HD view on patterning and differentiation. J Exp Bot.

[CR67] Reinhart BJ, Weinstein EG, Rhoades MW, Bartel B, Bartel DP (2002). MicroRNAs in plants. Genes Dev.

[CR68] Rivas FV, Tolia NH, Song JJ, Aragon JP, Liu J, Hannon GJ, Joshua-Tor L (2005). Purified Argonaute2 and an siRNA form recombinant human RISC. Nat Struct Mol Biol.

[CR69] Roccaro M, Somssich IE (2011). Chromatin immunoprecipitation to identify global targets of WRKY transcription factor family members involved in plant immunity. Methods Mol Biol.

[CR70] Saitou N, Nei M (1987). The neighbor-joining method: a new method for reconstructing phylogenetic trees. Mol Biol Evol.

[CR71] Sakamoto T, Morinaka Y, Ohnishi T, Sunohara H, Fujioka S, Ueguchi-Tanaka M, Mizutani M, Sakata K, Takatsuto S, Yoshida S, Tanaka H, Kitano H, Matsuoka M (2006). Erect leaves caused by brassinosteroid deficiency increase biomass production and grain yield in rice. Nat Biotechnol.

[CR72] Sato Y, Nishimura A, Ito M, Ashikari M, Hirano HY, Matsuoka M (2001). Auxin response factor family in rice. Genes Genet Syst.

[CR73] Schommer C, Palatnik JF, Aggarwal P, Chételat A, Cubas P, Farmer EE, Nath U, Weigel D (2008). Control of jasmonate biosynthesis and senescence by miR319 targets. PLoS Biol.

[CR74] Shen J, Xie K, Xiong L (2010). Global expression profiling of rice microRNAs by one-tube stem-loop reverse transcription quantitative PCR revealed important roles of microRNAs in abiotic stress responses. Mol Gen Genomics.

[CR75] Shi Z, Wang J, Wan X, Shen G, Wang X, Zhang J (2007). Over-expression of rice *OsAGO7* gene induces upward curling of the leaf blade that enhanced erect-leaf habit. Planta.

[CR76] Siegfried KR, Eshed Y, Baum SF, Otsuga D, Drews GN, Bowman JL (1999). Members of the *YABBY* gene family specify abaxial cell fate in *Arabidopsis*. Development.

[CR77] Sinclair TR, Sheehy JE (1999). Erect leaves and photosynthesis in rice. Science.

[CR78] Thompson JD, Higgins DG, Gibson TJ (1994). Clustal W: improving the sensitivity of progressive multiple sequence alignment through sequence weighting, position-specific gap penalties and weight matrix choice. Nucleic Acids Res.

[CR79] Toriba T, Harada K, Takamura A, Nakamura H, Ichikawa H, Suzaki T, Hirano HY (2007). Molecular characterization the *YABBY* gene family in *Oryza sativa* and expression analysis of *OsYABBY1*. Mol Gen Genomics.

[CR80] Toriba T, Suzaki T, Yamaguchi T, Ohmori Y, Tsukaya H, Hirano HY (2010). Distinct regulation of adaxial-abaxial polarity in anther patterning in rice. Plant Cell.

[CR81] Turchi L, Carabelli M, Ruzza V, Possenti M, Sassi M, Peñalosa A, Sessa G, Salvi S, Forte V, Morelli G, Ruberti I (2013). Arabidopsis HD-zip II transcription factors control apical embryo development and meristem function. Development.

[CR82] Vaucheret H, Vazquez F, Crete P, Bartel DP (2004). The action of *ARGONAUTE1* in the miRNA pathway and its regulation by the miRNA pathway are crucial for plant development. Genes Dev.

[CR83] Wang D, Pei K, Fu Y, Sun Z, Li S, Liu H, Tang K, Han B, Tao Y (2007). Genome-wide analysis of the *auxin response factors* (*ARF*) gene family in rice (*Oryza sativa*). Gene.

[CR84] Wang L, Gu X, Xu D, Wang W, Wang H, Zeng M, Chang Z, Huang H, Cui X (2011). miR396-targeted AtGRF transcription factors are required for coordination of cell division and differentiation during leaf development in *Arabidopsis*. J Exp Bot.

[CR85] Wang ST, Sun XL, Hoshino Y, Yu Y, Jia B, Sun ZW, Sun MZ, Duan XB, Zhu YM (2014). MicroRNA319 positively regulates cold tolerance by targeting *OsPCF6* and *OsTCP21* in rice (*Oryza sativa* L.). PLoS One.

[CR86] Wang W, Ye R, Xin Y, Fang X, Li C, Shi H, Zhou X, Qi Y (2011). An importin β protein negatively regulates microRNA activity in *Arabidopsis*. Plant Cell.

[CR87] Willemse MTM, Den Outer RW (1988). Stem anatomy and cell wall autofluorescence during growth of three maize (*Zea mays* L.) cultivars. Plant Biol.

[CR88] Williams L, Carles CC, Osmont KS, Fletcher JC (2005). A database analysis method identifies an endogenous trans-acting short-interfering RNA that targets the *Arabidopsis ARF2*, *ARF3*, and *ARF4* genes. Proc Natl Acad Sci U S A.

[CR89] Williams L, Grigg SP, Xie M, Christensen S, Fletcher JC (2005). Regulation of *Arabidopsis* shoot apical meristem and lateral organ formation by microRNA *miR166g* and its *AtHD-ZIP* target genes. Development.

[CR90] Wu J, Yang R, Yang Z, Yao S, Zhao S, Wang Y, Li P, Song X, Jin L, Zhou T, Lan Y, Xie L, Zhou X, Chu C, Qi Y, Cao X, Li Y (2017). ROS accumulation and antiviral defence control by microRNA528 in rice. Nat Plants.

[CR91] Wu J, Yang Z, Wang Y, Zheng L, Ye R, Ji Y, Zhao S, Ji S, Liu R, Xu L, Zheng H, Zhou Y, Zhang X, Cao X, Xie L, Wu Z, Qi Y, Li Y (2015). Viral-inducible Argonaute18 confers broad-spectrum virus resistance in rice by sequestering a host microRNA. ELife.

[CR92] Wu L, Zhang Q, Zhou H, Ni F, Wu X, Qi Y (2009). Rice microRNA effector complexes and targets. Plant Cell.

[CR93] Xia R, Xu J, Meyers BC (2017). The emergence, evolution, and diversification of the miR390-*TAS3*-*ARF* pathway in land plants. Plant Cell.

[CR94] Xiang J, Zhang G, Qian Q, Xue H (2012). *SEMI-ROLLED LEAF1* encodes a putative glycosylphosphatidylinositol-anchored protein and modulates rice leaf rolling by regulating the formation of bulliform cells. Plant Physiol.

[CR95] Yamaguchi T, Nagasawa N, Kawasaki S, Matsuoka M, Nagato Y, Hirano HY (2004). The *YABBY* gene *DROOPING LEAF* regulates carpel specification and midrib development in *Oryza sativa*. Plant Cell.

[CR96] Yamaguchi T, Nukazuka A, Tsukaya H (2012). Leaf adaxial-abaxial polarity specification and lamina outgrowth: evolution and development. Plant Cell Physiol.

[CR97] Yang C, Li D, Liu X, Ji C, Hao L, Zhao X, Li X, Chen C, Cheng Z, Zhu L (2014). OsMYB103L, an R2R3-MYB transcription factor, influences leaf rolling and mechanical strength in rice (*Oryza sativa* L.). BMC Plant Biol.

[CR98] Yang C, Li D, Mao D, Liu X, Ji C, Li X, Zhao X, Cheng Z, Chen C, Zhu L (2013). Overexpression of microRNA319 impacts leaf morphogenesis and leads to enhanced cold tolerance in rice (*Oryza sativa* L.). Plant Cell Environ.

[CR99] Yang JW, Fu JX, Li J, Cheng XL, Li F, Dong JF, Liu ZL, Zhuang CX (2014). A novel co-immunoprecipitation protocol based on protoplast transient gene expression for studying protein-protein interactions in rice. Plant Mol Biol Rep.

[CR100] Yang L, Huang W, Wang H, Cai R, Xu Y, Huang H (2006). Characterizations of a hypomorphic *argonaute1* mutant reveal novel *AGO1* functions in *Arabidopsis* lateral organ development. Plant Mol Biol.

[CR101] Yang SQ, Li WQ, Miao H, Gan PF, Qiao L, Chang YL, Shi CH, Chen KM (2016). *REL2*, a gene encoding an unknown function protein which contains DUF630 and DUF632 domains controls leaf rolling in rice. Rice.

[CR102] Yang T, Wang Y, Teotia S, Zhang Z, Tang G (2018). The making of leaves: how small RNA networks modulate leaf development. Front Plant Sci.

[CR103] Ye R, Wang W, Iki T, Liu C, Wu Y, Ishikawa M, Zhou X, Qi Y (2012). Cytoplasmic assembly and selective nuclear import of *Arabidopsis* ARGONAUTE4/siRNA complexes. Mol Cell.

[CR104] Ye Y, Liu B, Zhao M, Wu K, Cheng W, Chen X, Liu Q, Liu Z, Fu X, Wu Y (2015). CEF1/OsMYB103L is involved in GA-mediated regulation of secondary wall biosynthesis in rice. Plant Mol Biol.

[CR105] Yuan LP (1997). Hybrid rice breeding for super high yield. Hybrid Rice.

[CR106] Zhang DB, Wilson ZA (2009). Stamen specification and anther development in rice. Chin Sci Bull.

[CR107] Zhang G, Xu Q, Zhu X, Qian Q, Xue H (2009). SHALLOT-LIKE1 is a KANADI transcription factor that modulates rice leaf rolling by regulating leaf abaxial cell development. Plant Cell.

[CR108] Zhang J, Zhang H, Srivastava AK, Pan Y, Bai J, Fang J, Shi H, Zhu JK (2018). Knockdown of rice microRNA166 confers drought resistance by causing leaf rolling and altering stem xylem development. Plant Physiol.

[CR109] Zhao S, Zhao L, Liu F, Wu Y, Zhu Z, Sun C, Tan L (2016). *NARROW AND ROLLED LEAF 2* regulates leaf shape, male fertility, and seed size in rice. J Integr Plant Biol.

[CR110] Zheng S, Li J, Ma L, Wang H, Zhou H, Ni E, Jiang D, Liu Z, Zhuang C (2019). *OsAGO2* controls ROS production and the initiation of tapetal PCD by epigenetically regulating OsHXK1 expression in rice anthers. Proc Natl Acad Sci U S A.

[CR111] Zhong R, Ye Z (2007). Regulation of cell wall biosynthesis. Curr Opin Plant Biol.

[CR112] Zhou C, Han L, Fu C, Wen J, Cheng X, Nakashima J, Ma J, Tang Y, Tan Y, Tadege M, Mysore KS, Xia G, Wang ZY (2013). The *trans*-acting short interfering RNA3 pathway and NO APICAL MERISTEM antagonistically regulate leaf margin development and lateral organ separation, as revealed by analysis of an *argonaute7*/*lobed leaflet1* mutant in *Medicago truncatula*. Plant Cell.

[CR113] Zhou H, Zhou M, Yang Y, Li J, Zhu L, Jiang D, Dong J, Liu Q, Gu L, Zhou L, Feng M, Qin P, Hu X, Song C, Shi J, Song X, Ni E, Wu X, Deng Q, Liu Z, Chen M, Liu YG, Cao X, Zhuang C (2014). RNase Z^S1^ processes *Ub*_*L40*_ mRNAs and controls thermosensitive genic male sterility in rice. Nat Commun.

[CR114] Zhou Y, Honda M, Zhu H, Zhang Z, Guo X, Li T, Li Z, Peng X, Nakajima K, Duan L, Zhang X (2015). Spatiotemporal sequestration of mir165/166 by *Arabidopsis* Argonaute10 promotes shoot apical meristem maintenance. Cell Rep.

[CR115] Zhu H, Hu F, Wang R, Zhou X, Sze SH, Liou LW, Barefoot A, Dickman M, Zhang X (2011). *Arabidopsis* Argonaute10 specifically sequesters miR166/165 to regulate shoot apical meristem development. Cell.

[CR116] Zou LP, Sun XH, Zhang ZG, Liu P, Wu JX, Tian CJ, Qiu JL, Lu TG (2011). Leaf rolling controlled by the homeodomain leucine zipper class IV gene *Roc5* in rice. Plant Physiol.

