# Supplementary material for: Overexpression of OsAGO1b Induces Adaxially Rolled Leaves by Affecting Leaf Abaxial Sclerenchymatous Cell Development in Rice
Source: Rice (N Y). 2019 Aug 8;12:60. doi: 10.1186/s12284-019-0323-9 (PMC6687834; doi:10.1186/s12284-019-0323-9)
Supplement: Supplementary file 1 — Table S1. Primers used in this study. (DOCX 27 kb) [file 12284_2019_323_MOESM1_ESM.docx]

**Additional file 1: Table S1. Primers used in this study**

| **Primer name** | **Sequence (from 5' to 3')** | **Purpose** |
| --- | --- | --- |
| AGO1b-OF | aaaaAAGCTTgcaagcgctcgtc gtctttc (*Hin*d III) | Used for *OsAGO1b* overexpression vector construction |
| AGO1b-OR | aaaaACTAGTcaacgacagagtgcaagtgct (*Spe* I) |  |
| AGO1b-RF | aaaaGGATCCcgtgtcatgttttactgctaag (*Bam* HI) | Used for *OsAGO1b* RNAi vector construction |
| AGO1b-RR | aaaaAAGCTTtgcagcgtcacaaacacaaat (*Hin*d III) |  |
| RNAi-Mlu | caccctgACGCGTggtgttacttctgaagagg (*Mlu* I) | pRNAi-Ubi universal primer used for sense fragment amplification and antisense fragment cloning |
| RNAi-Pst | actagaaCTGCAGcctcagatctaccatggtcg (*Pst* I) |  |
| UbiF | aaaaGGATCCagtgc agcgtgaccc ggtcg (*Bam* HI) | Used for maize *ubi* promoter cloning into pSK |
| UbiR | aaaaAAGCTTcagaagtaacaccaaacaacagg (*Hin*d III) |  |
| AGO1a-OF | aaaaCCCGGG ggagaaagtttccactctgtctg (*Sma* I） | Used for *OsAGO1a* overexpression vector construction |
| AGO1a-OR | aaaaACTAGTcgtacacacactataggtcgttc-3’ (*Spe* I) |  |
| AGO1a-3RF | aaaaGGATCCgtcatgttctactgctaagt (*Bam* HI) | Used for *OsAGO1a* RNAi fragment amplification and RNAi vector construction |
| AGO1a-3RR | aaaaAAGCTTggctaataatccttgcgaat (*Hind* III) |  |
| AGO1d-OF | aaaaCCCGGGccttctactagtactagtacatc (*Sma* I) | Used for *OsAGO1d* overexpression vector construction |
| AGO1d-OR | aaaaAAGCTTgcttcagaagtcttgcatacttc (*Hin*d III) |  |
| AGO1d-3RF | aaaaGGATCC ttctactact gcttcgcttg (*Bam* HI) | Used for *OsAGO1d* RNAi fragment amplification and RNAi vector construction |
| AGO1d-3RR | aaaa AAGCTT cgaaaatccaaatgcaaacaag (*Hin*d III) |  |
| AGO1c-OF | aaaaAAGCTTaactctccatccatcgatcgatc (*Hin*d III) | Used for *OsAGO1c* overexpression vector construction |
| AGO1c-OR | aaaaACGCGTtacaaccatcataggcaggttgc (*Mlu* I) |  |
| AGO1c-RF | aaaaGGATCCtcatgttctactgctgaagcta (*Bam* HI) | Used for *OsAGO1c* RNAi fragment amplification and RNAi vector construction |
| AGO1c-RR | aaaaAAGCTTctcgaatcataactacagacatg (*Hin*d III) |  |
| AGO1b-YFP-F | aaaaGGGCCCctggtgaagaagaaaagaac (*Sma* I) | Used for YFP-OsAGO1b transient expression vector construction |
| AGO1b-YFP-R | aaaaAAGCTTgcagtaaaacatgacacgc (*Hin*d III) |  |
| HPT-F | catcgaaattgccgtcaacc | Use for *Hyg* probe fragment amplification for Southern blot |
| HPT-R | gctttcagttcgatgtagg |  |
| AGO1b-qF | accagcgtactgttgataga | *OsAGO1b* qRT-PCR |
| AGO1b-qR | caagttgttcgtgagggttt |  |
| SLL1-qF | cggcaacaccagcaccacca | *OsSLL1* qRT-PCR |
| SLL1-qR | tctgcacggttccaagttctca |  |
| SRL2-qF | tcctcgtgtcttaggtgttg | *OsSRL2* qRT-PCR |
| SRL2-qR | gactggtcattgtgccgtat |  |
| C4H-qF | ggcgagatcaaccacgacaacg | *OsC4H* qRT-PCR |
| C4H-qR | gcaaccgcagcgtctccttca |  |
| 4CL-qF | catcgtggacaggctcaa | *Os4CL3* qRT-PCR |
| 4CL-qR | aaggcgactggcacttca |  |
| TED2-qF | aaagcaatccaagcagccgaagacg | *OsTED2* qRT-PCR |
| TED2-qR | ccacgcggcatcagacgctccattg |  |
| PI-qF | atcaagccggaggtcgccatc | *OsPI* qRT-PCR |
| PI-qR | caaggcagcgtgtaatctcc |  |
| CP-qF | ccaacaggttcgccgacctcac | *OsCP* qRT-PCR |
| CP-qR | ccgcacgagccttggtccttga |  |
| YAB1-qF | gtcggtccagtttacatcgg | *OsYABBY1* qRT-PCR |
| YAB1-qR | tgcccacatctaacagtcacaa |  |
| YAB2-qF | gcatttcccgaacatcca | *OsYABBY2* qRT-PCR |
| YAB2-qR | caatacagcctttgaacctttt |  |
| YAB4-qF | gacgccgagcctactgatgga | *OsYABBY4* qRT-PCR |
| YAB4-qR | ccggttgtacgcagatgggact |  |
| YAB6-qF | cccgacattagccacagg | *OsYABBY6* qRT-PCR |
| YAB6-qR | acgagcttcttgccaccc |  |
| YAB7-qF | gcggagacgatagggaggga | *OsYABBY7* qRT-PCR |
| YAB7-qR | gacgaaacagttgtaggcagacg |  |
| ARF2-qF | ggcgatgaagtggattgtg | *OsARF2* qRT-PCR |
| ARF2-qR | tatcaacgaccctgccaac |  |
| ARF3-qF | gcggagggcgaggtatttg | *OsARF3* qRT-PCR |
| ARF3-qR | agcagcccgacgaggaacag |  |
| ARF4-qF | gagcccttcagagttcatt | *OsARF4* qRT-PCR |
| ARF4-qR | gtgcctcttctccttcaaa |  |
| ARF14-qF | ccctcgtcggttctgatgt | *OsARF14* qRT-PCR |
| ARF14-qR | tgatttcgctcggcgtttg |  |
| ARF15-qF | ggaggttccgccatattta | *OsARF15* qRT-PCR |
| ARF15-qR | cactttctgtagccactgc |  |
| HB1-qF | cgccttacaggacctcacat | *OsHB1* qRT-PCR |
| HB1-qR | gcaagcactttccaagccac |  |
| HB2-qF | gctatttctccttctcggtctg | *OsHB2* qRT-PCR |
| HB2-qR | agcatcatctgcctgcctaa |  |
| HB3-qF | gcggcaatggctaggagtta | *OsHB3* qRT-PCR |
| HB3-qR | aatcggcatcttcggtatca |  |
| HB4-qF | cagattgaaacaaagaaccctcc | *OsHB4* qRT-PCR |
| HB4-qR | atcagcgtcgtctccagcat |  |
| HB5-qF | gacgctgcccagatggattt | *OsHB5* qRT-PCR |
| HB5-qR | acactggcatcgccttgaga |  |
| actin-qF | cacattccagcagatgtgga | *OsActin1* qRT-PCR |
| actin-qR | gcgataacagctcctcttgg |  |
| miR166-stemloop | gtcgtatccagtgcagggtccgaggtattcgcactggatacgacgggaa | miR166 reverse transcript |
| miR319-stemloop | gtcgtatccagtgcagggtccgaggtattcgcactggatacgacgggagc | miR319 reverse transcript |
| miR390-stemloop | gtcgtatccagtgcagggtccgaggtattcgcactggatacgacggcgct | miR390 reverse transcript |
| miR396-stemloop | gtcgtatccagtgcagggtccgaggtattcgcactggatacgaccagttc | miR396 reverse transcript |
| TAS3-siRNA-D6(+)-stemloop | gtcgtatccagtgcagggtccgaggtattcgcactggatacgactgggtc | *TAS3*-tasiRNA-D6(+) reverse transcript |
| TAS3-siRNA-D7(+)-stemloop | gtcgtatccagtgcagggtccgaggtattcgcactggatacgacaaggtc | *TAS3*-tasiRNA-D7(+) reverse transcript |
| miR166-qF | atgcggtcggaccaggcttca | miR166 qRT-PCR |
| miR319-qF | tcggcggttggactgaagggt | miR319 qRT-PCR |
| miR390-qF | tggcggaagctcaggagggat | miR390 qRT-PCR |
| miR396-qF | cggcggttccacagctttctt | miR396 qRT-PCR |
| TAS3-siRNA-D6(+)-qF | cgggcgttcttgaccttgtaa | *TAS3*-tasiRNA-D6(+) qRT-PCR |
| TAS3-siRNA-D7(+)-qF | cgggcgttcttgaccttgcaa | *TAS3*-tasiRNA-D6(+) qRT-PCR |
| small RNA universal primer R | ccagtgcagggtccgaggt | universal reverse primer for small RNA qRT-PCR |
| 5S-rRNA-qF | aaacacccgatcccattcc | 5S ribosomal RNA gene *rrn5* qRT-PCR |
| 5S-rRNA-qR | ggctttgaccatgtctccc |  |
| OsPCF5-F | cctacctgttcaacgtgtcg | *OsPCF5* qRT-PCR |
| OsPCF5-R | tcatctggttgtcggcttc |  |
| OsPCF6-F | cacctttccagcaagagctcac | *OsPCF6* qRT-PCR |
| OsPCF6-R | ggccaaaaaacagcatagcaga |  |
| OsPCF7-F | tacgggttcggcaacacta | *OsPCF7* qRT-PCR |
| OsPCF7-R | acttgacgctgtcggagact |  |
| OsPCF8-F | cacatccgagaccagcaagag | *OsPCF8* qRT-PCR |
| OsPCF8-R | cgcattggactgaagtgtg |  |
| OsTCP21-F | cctcgggtcttgtgggtgt | *OsPCF21* qRT-PCR |
| OsTCP21-R | gaggaacggaatgttgggc |  |
| OsAGO7-F | ttatggctggtgaactgct | *OsAGO7* qRT-PCR |
| OsAGO7-R | atgcctcttctgaactacgat |  |
| OsPNH1-F | atgggaggaagaaacaccg | *OsPNH1* qRT-PCR |
| OsPNH1-R | gcaatggatgggctagagt |  |
| ISH-AGO1b-NF-A | caaaccctcacgaacaact | New primers used for *OsAGO1b* *in situ* hydridization antisense probe amplification |
| ISH-AGO1b-NR-A | tgtaatacgactcactatagggcaagcacaacaaaggtccaa |  |
| ISH-AGO1b-NF-S | tgtaatacgactcactatagggccaaaccctcacgaacaact | New primers used for *OsAGO1b* *in situ* hydridization sense probe amplification |
| ISH-AGO1b-NF-S | aagcacaacaaaggtccaa |  |
| ISH-OSHB3-NF-A | caatagctccttctcgacttgg | Primers used for *OSHB3* *in situ* hydridization antisense probe amplification |
| ISH-OSHB3-NR-A | tgtaatacgactcactatagggcgttcatccacacagcagcagaa |  |
| ISH-OSHB3-NF-S | tgtaatacgactcactatagggccaatagctccttctcgacttgg | primers used for *OSHB3* *in situ* hydridization sense probe amplification |
| ISH-OSHB3-NF-S | gttcatccacacagcagcagaa |  |
| ISH-ARF4-NF-A | gcctgcttcatcacctcct | primers used for *OsARF4* *in situ* hydridization antisense probe amplification |
| ISH-ARF4-NR-A | tgtaatacgactcactatagggctgcggtctaatcactcttgg |  |
| ISH-ARF4-NF-S | tgtaatacgactcactatagggcgcctgcttcatcacctcct | primers used for *OsARF4* *in situ* hydridization sense probe amplification |
| ISH-ARF4-NF-S | tgcggtctaatcactcttgg |  |

Note: The capital letters in primer sequence represent the enzyme sites.
