# Supplementary material for: Overexpression of OsAGO1b Induces Adaxially Rolled Leaves by Affecting Leaf Abaxial Sclerenchymatous Cell Development in Rice
Source: Rice (N Y). 2019 Aug 8;12:60. doi: 10.1186/s12284-019-0323-9 (PMC6687834; doi:10.1186/s12284-019-0323-9)
Supplement: Supplementary file 2 — Table S2. Information of the construction of overexpression and RNAi vectors for rice four AGO1 genes. (DOCX 15 kb) [file 12284_2019_323_MOESM2_ESM.docx]

**Additional file 2: Table S2. Information of the construction of overexpression and RNAi vectors for rice four *AGO1* genes**

| **Construct names** | **Vectors** | **Fragment length** | **Digestion sites** | **Primer pairs** | **Accession numbers** |
| --- | --- | --- | --- | --- | --- |
| *OsAGO1a*-Overexpression | pSK-Ubi pCAMBIA1390 | 3474 bp | *Bam* HI+*Sma* I for *ubi* promoter  *Sma* I+*Spe* I for target gene | AGO1a-OF  AGO1a-OR | Q6EU14 LOC_Os02g45070 Os02g0672200 |
| *OsAGO1a*-RNAi | pRNAi-Ubi | 342 bp | *Bam* HI+*Hin*d III for sense orientation  *Mlu* I+*Pst* I for antisense orientation | AGO1a-3RF  AGO1a-3RR  RNAi-Mlu  RNAi-Pst |  |
| *OsAGO1b*-Overexpression | pSK-Ubi pCAMBIA1380 | 3399 bp | *Bam* HI+*Hind* III for *ubi* promoter  *Hin*d III+*Spe* I for target gene | AGO1b-OF  AGO1b-OR | Q7XSA2  LOC_Os04g47870  Os04g0566500 |
| *OsAGO1b*-RNAi | pRNAi-Ubi | 191 bp | *Bam* HI+*Hin*d III for sense orientation  *Mlu* I+*Pst* I for antisense orientation | AGO1b-RF  AGO1b-RR  RNAi-Mlu  RNAi-Pst |  |
| *OsAGO1c*-Overexpression | pOx | 3185 bp | *Hin*d III+*Mlu* I for target gene | AGO1c-OF  AGO1c-OR | Q6K972  LOC_Os02g58490  Os02g0831600 |
| *OsAGO1c*-RNAi | pRNAi-Ubi | 259 bp | *Bam* HI+*Hin*d III for sense orientation  *Mlu* I+*Pst* I for antisense orientation | AGO1c-RF  AGO1c-RR  RNAi-Mlu  RNAi-Pst |  |
| *OsAGO1d*-Overexpression | pSK-ubi pCAMBIA1380 | 3358 bp | *Sma* I+*Hin*d III for target gene into pSK-ubi  *Hin*d III+*Bam* HI for *ubi*+target gene into 1380 | AGO1d-OF  AGO1d-OR | Q5Z5B2  LOC_Os06g51310  Os06g0729300 |
| *OsAGO1d*-RNAi | pRNAi-Ubi | 300 bp | *Bam* HI+*Hin*d III for sense orientation  *Mlu* I+*Pst* I for antisense orientation | AGO1d-3RF  AGO1d-3RR  RNAi-Mlu  RNAi-Pst |  |

Note: All the inserted fragments were driven by maize *ubiquitin* (*ubi*) promoter. The pOx overexpression vector was modified from pRNAi-Ubi (see Materials and methods) RNAi vector designed by Dr. Yaoguang Liu’s lab in SCAU. The multiple cloning site (MCS) of pOx include maize *ubi* promoter without ATG start coden followed successively by digestion sites *Pst* I, *Kpn* I, *Hind* III, *Spe* I, *Mlu* I, and *Bam* HI.
