# Supplementary material for: Overexpression of OsAGO1b Induces Adaxially Rolled Leaves by Affecting Leaf Abaxial Sclerenchymatous Cell Development in Rice
Source: Rice (N Y). 2019 Aug 8;12:60. doi: 10.1186/s12284-019-0323-9 (PMC6687834; doi:10.1186/s12284-019-0323-9)
Supplement: Supplementary file 3 — Table S3. Information of plant AGO1 proteins used for phylogenetic analysis. (DOCX 12 kb) [file 12284_2019_323_MOESM3_ESM.docx]

**Additional file 3: Table S3.** **Information of plant AGO1 proteins used for phylogenetic analysis**

| **Protein names** | **Accession numbers** | **Species origin** | **Species names** |
| --- | --- | --- | --- |
| OsAGO1a | Q6EU14 | rice | *Oryza sativa* L. |
| OsAGO1b | Q7XSA2 | rice | *Oryza sativa* L. |
| OsAGO1c | Q6K972 | rice | *Oryza sativa* L. |
| OsAGO1d | Q5Z5B2 | rice | *Oryza sativa* L. |
| ZmAGO1a | AIN41713 | maize | *Zea mays* L. |
| ZmAGO1b | XP_008663491.1 | maize | *Zea mays* L. |
| ZmAGO1c | XP_008668223 | maize | *Zea mays* L. |
| ZmAGO1d | XP_008645117 | maize | *Zea mays* L. |
| SiAGO1b | XP_004976514 | millet | *Setaria italica* |
| SiAGO1b | XP_012700084 | millet | *Setaria italica* |
| SiAGO1c | XP_004954525 | millet | *Setaria italica* |
| SiAGO1d | XP_012700523 | millet | *Setaria italica* |
| TaAGO1 | AGB34310 | wheat | *Triticum aestivum* L. |
| AtAGO1 | NP_849784 | Arabidopsis | *Arabidopsis thaliana* |
| GmAGO1 | XP_003534084 | soybean | *Glycine max* |
| MtAGO1 | XP_013452827 | alfafa | *Medicago truncatula* |
| SlAGO1a | NP_001266057 | tomato | *Solanum lycopersicum* |
| SlAGO1b | NP_001266261 | tomato | *Solanum lycopersicum* |
| NtAGO1 | NP_001312434 | tobacco | *Nicotiana tabacum* |
