# Supplementary figures and images for: Overexpression of OsAGO1b Induces Adaxially Rolled Leaves by Affecting Leaf Abaxial Sclerenchymatous Cell Development in Rice
Source: Rice (N Y). 2019 Aug 8;12:60. doi: 10.1186/s12284-019-0323-9 (PMC6687834; doi:10.1186/s12284-019-0323-9)

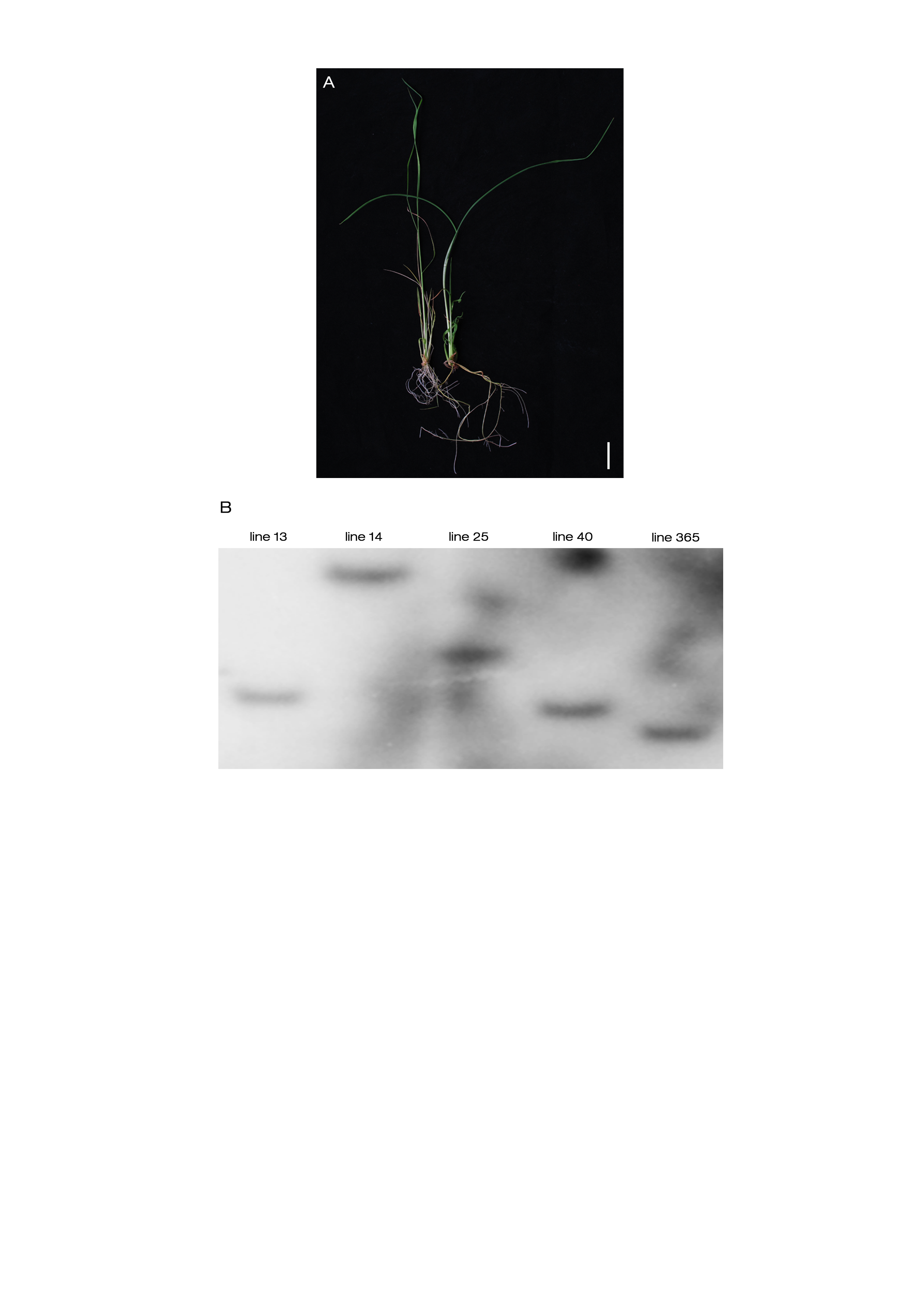

Supplement: Supplementary file 4 — Figure S1. Characterization of T0 transgenic plants of the OE-AGO1b lines. (A) Regenerated seedlings for the control empty vector pCAMBIA1380 (left) and OsAGO1b-overexpression construct (right). Scale bar = 1 cm. (B) Detection of T-DNA insertion numbers of OsAGO1b-overexpression lines using Southern blot analysis. Transgenic lines 25, 40, 365 containing a single copy T-DNA were chosen for further study. OE-AGO1b, OsAGO1b-overexpression line. (TIF 5135 kb) [file 12284_2019_323_MOESM4_ESM.tif]

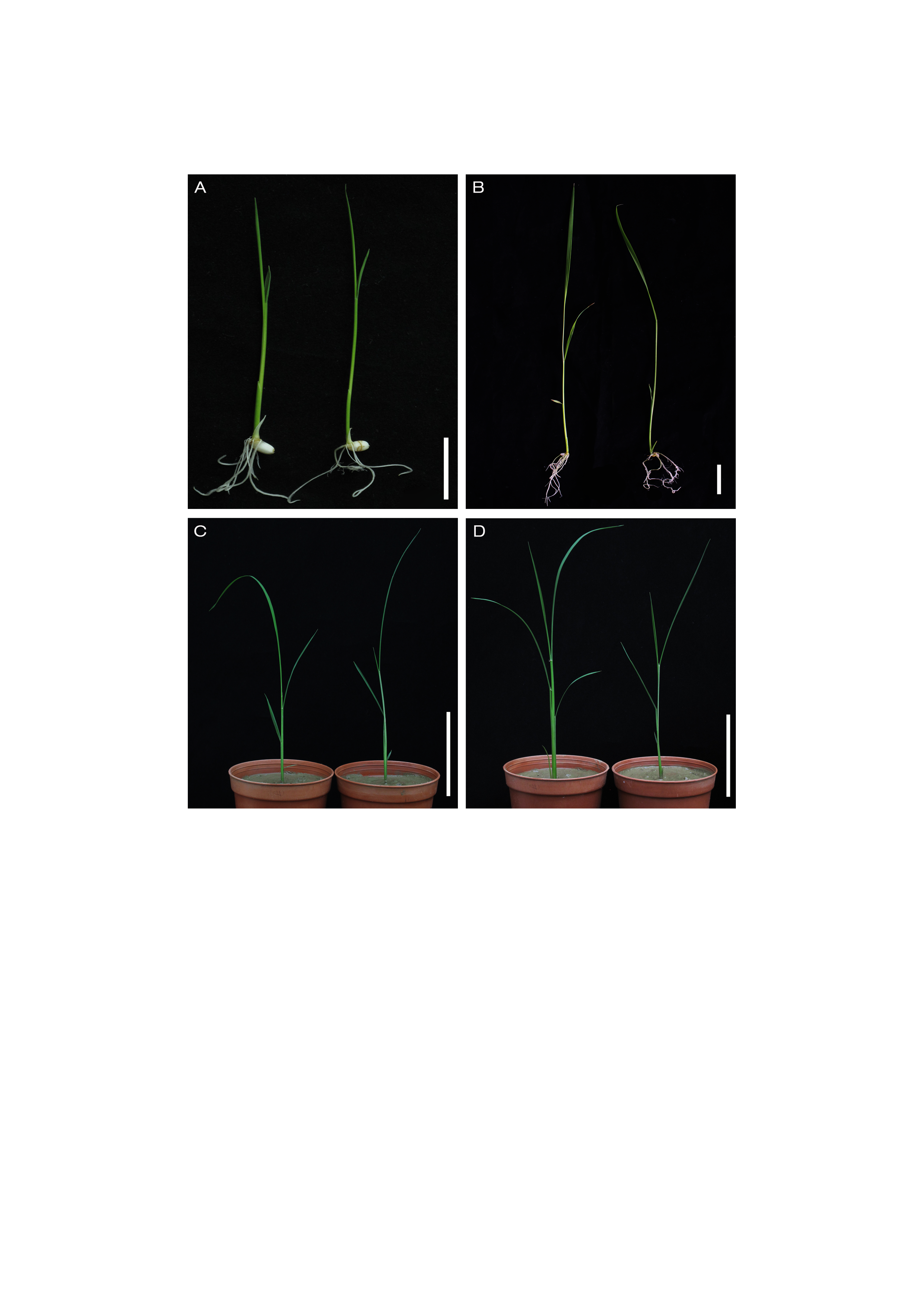

Supplement: Supplementary file 5 — Figure S2. Young leaf development of the OE-AGO1b line and ZH11. (A-D) indicates 7-day-old, 14-day-old, 21-day-old, and 28-day-old seedlings after germination. Scale bars = 2 cm for (A) and (B). Scale bars = 10 cm for (C) and (D). The OsAGO1b-overexpression line displayed adaxially rolled leaves from the fourth leaf stage. OE-AGO1b, OsAGO1b-overexpression line; ZH11, wild type Zhonghua 11. (TIF 9073 kb) [file 12284_2019_323_MOESM5_ESM.tif]

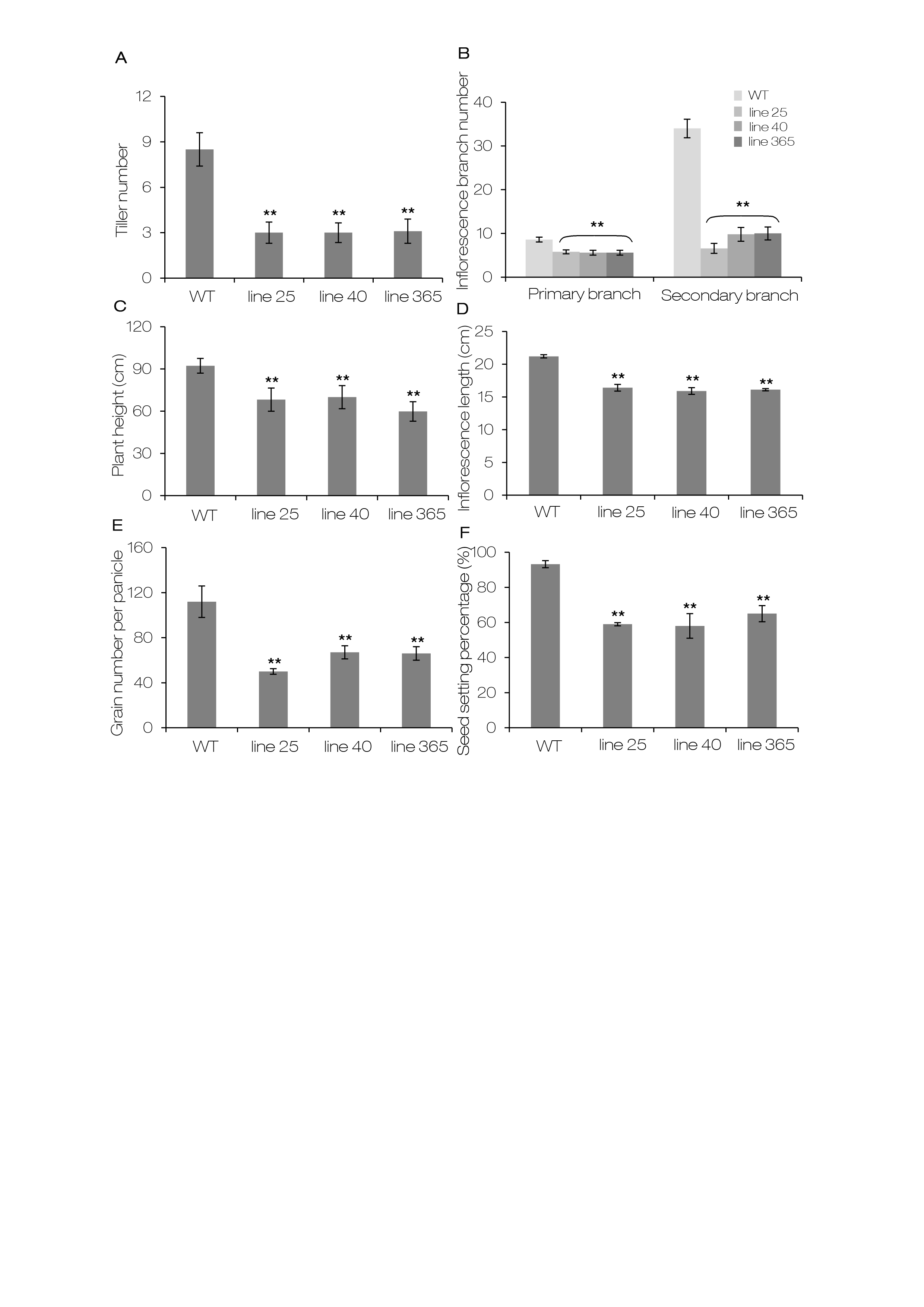

Supplement: Supplementary file 6 — Figure S3. Agronomic traits of ZH11 and OE-AGO1b lines. (A-F) Tiller number, inflorescence branch number, plant height, inflorescence length, grain number per panicle, and seed setting percentage of ZH11 (WT) and OE-AGO1b (line 25, line 40, line 365), respectively. Results are shown as the mean ± SD in (A)-(F) (n = 10). ** P < 0.01 (one-way ANOVA). OE-AGO1b, OsAGO1b-overexpression line; ZH11, wild type Zhonghua 11. (TIF 1467 kb) [file 12284_2019_323_MOESM6_ESM.tif]

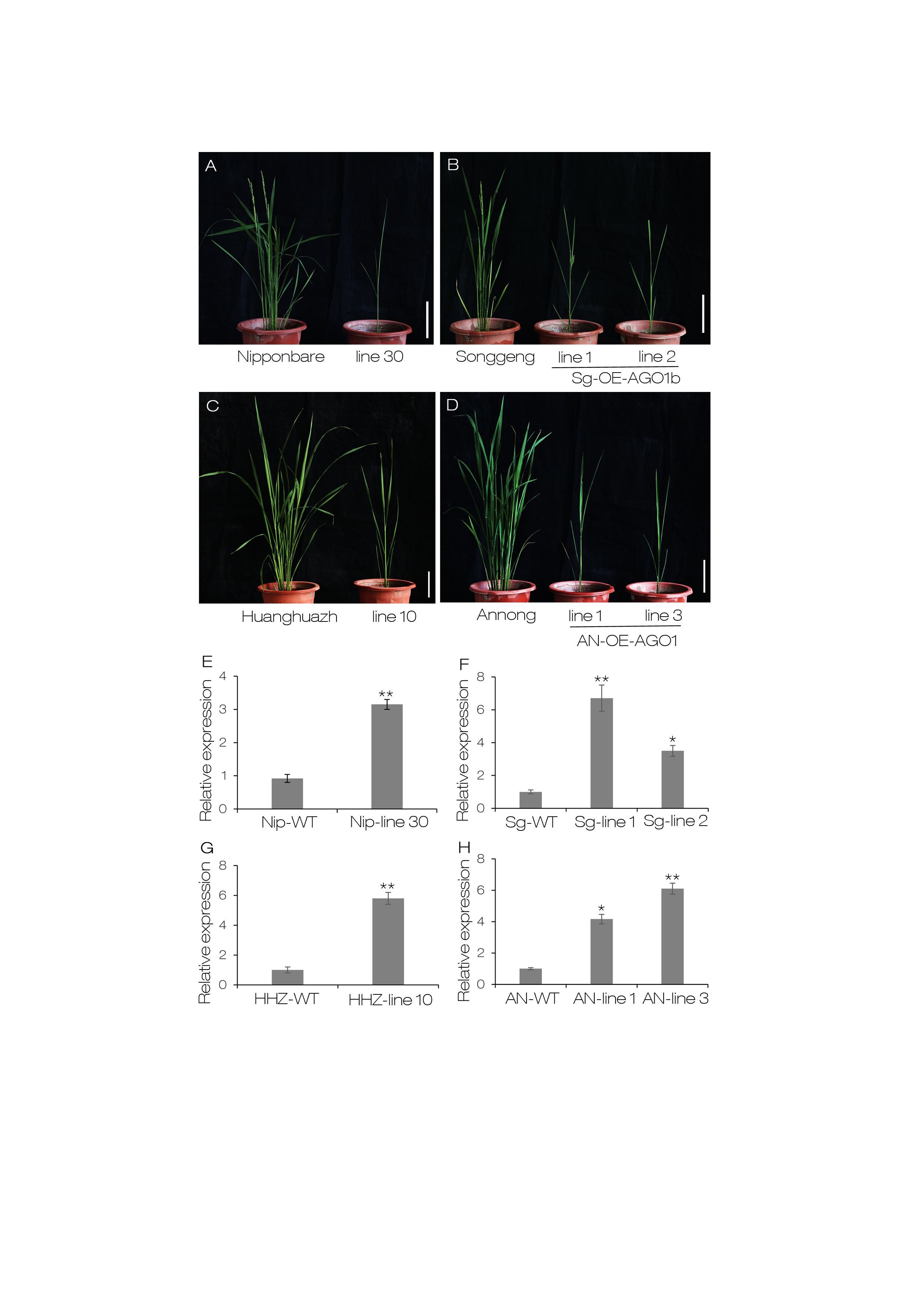

Supplement: Supplementary file 7 — Figure S4. Phenotypes of OE-AGO1b lines for different rice varieties. (A-D) Wild type and OE-AGO1b plants at the booting stage or heading stage for Nipponbare, Songgeng, Huanghuazhan and Annong. All the plants were geminated and planted at the same time under the same conditions. Scale bars = 15 cm. (E-H) Relative expression of OsAGO1b in OE-AGO1b plants of Nipponbare (Nip), Songgeng (Sg), Huanghuazhan (HHZ) and Annong (AN) backgrounds. Flag leaves at the booting stage were used for RNA extraction and qRT-PCR analysis. Means ± SD are presented in (E-H) (n = 3). * P < 0.05, ** P < 0.01 (one-way ANOVA). OE-AGO1b, OsAGO1b-overexpression; WT, wild type. (TIF 8027 kb) [file 12284_2019_323_MOESM7_ESM.tif]

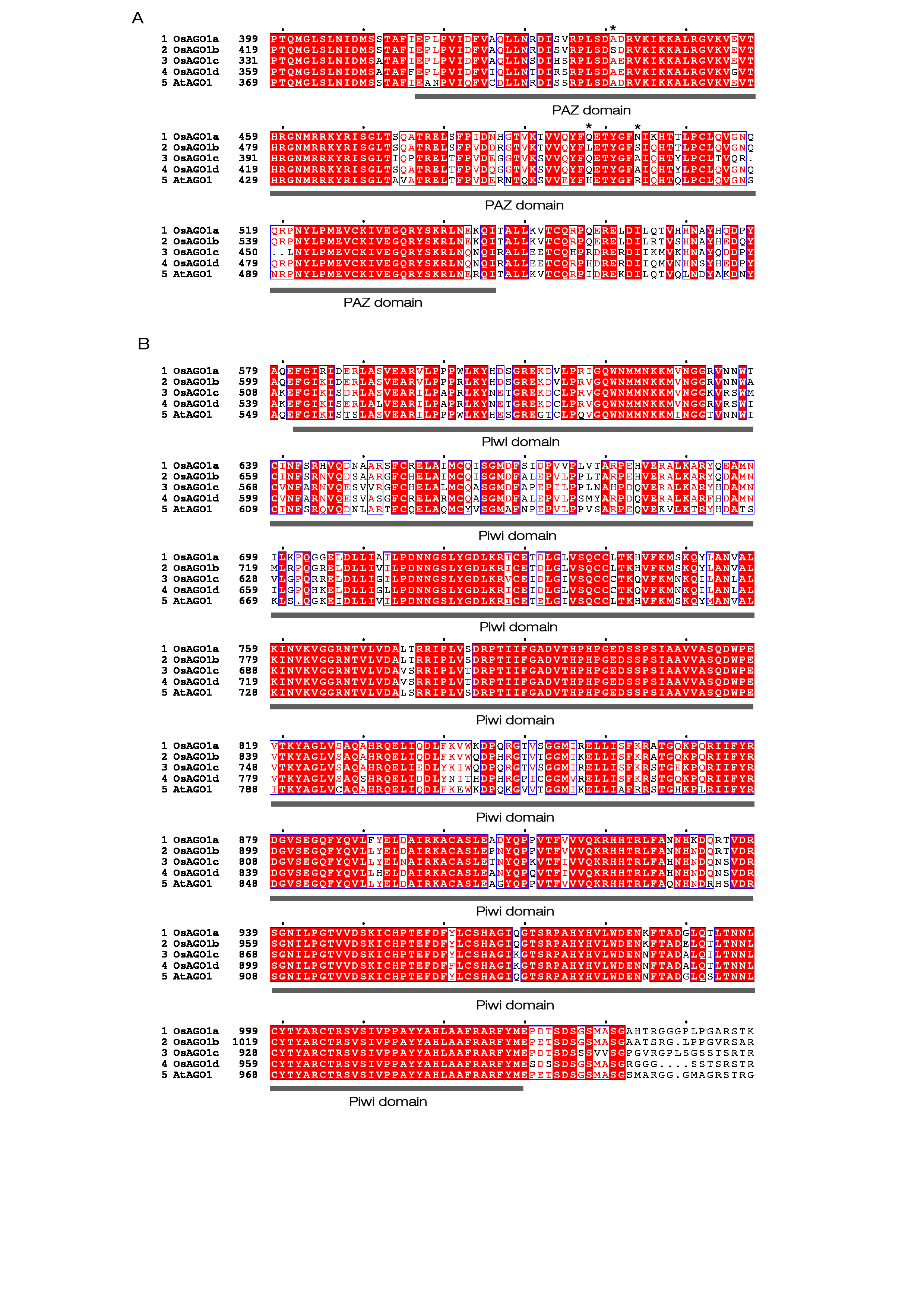

Supplement: Supplementary file 8 — Figure S5. Amino acid sequences of the PAZ and Piwi domains of OsAGO1s and AtAGO1. Alignment of amino acid sequences of the PAZ domain (A) the Piwi domains (B) of OsAGO1s and AtAGO1. “*” indicated the polymorphic amino acid residues specific to OsAGO1b. (TIF 3570 kb) [file 12284_2019_323_MOESM8_ESM.tif]

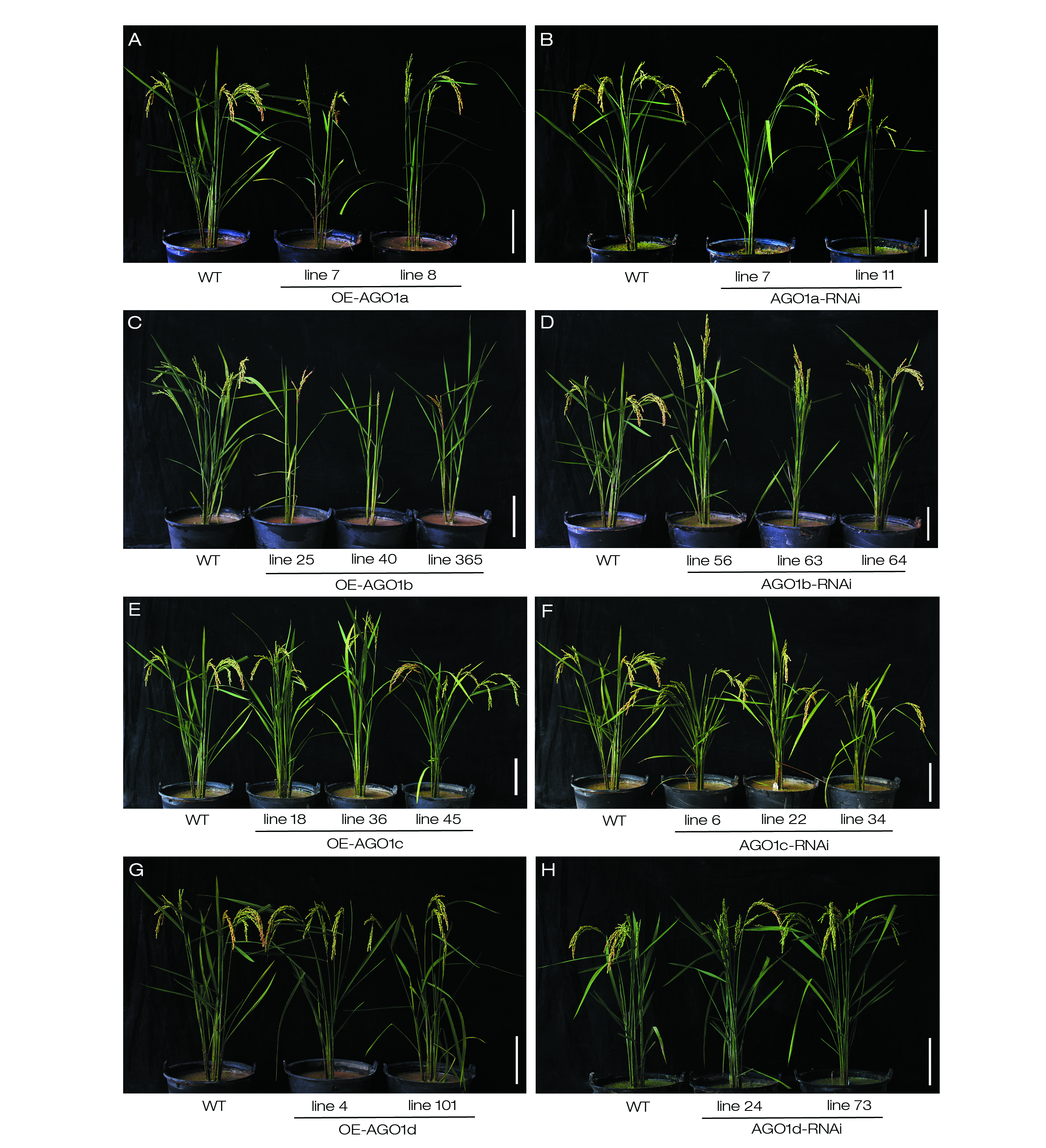

Supplement: Supplementary file 9 — Figure S6. Phenotypes of four rice AGO1-overexpression and RNAi transgenic plants. (A, C, E, G) Morphologies of lines overexpressing OsAGO1a, OsAGO1b, OsAGO1c and OsAGO1d at the mature stage. (B, D, F, H) Morphologies of RNAi lines for OsAGO1a, OsAGO1b, OsAGO1c and OsAGO1d at the mature stage. Scale bars = 15 cm. WT, wild type Zhonghua 11; OE, overexpression. (TIF 23721 kb) [file 12284_2019_323_MOESM9_ESM.tif]

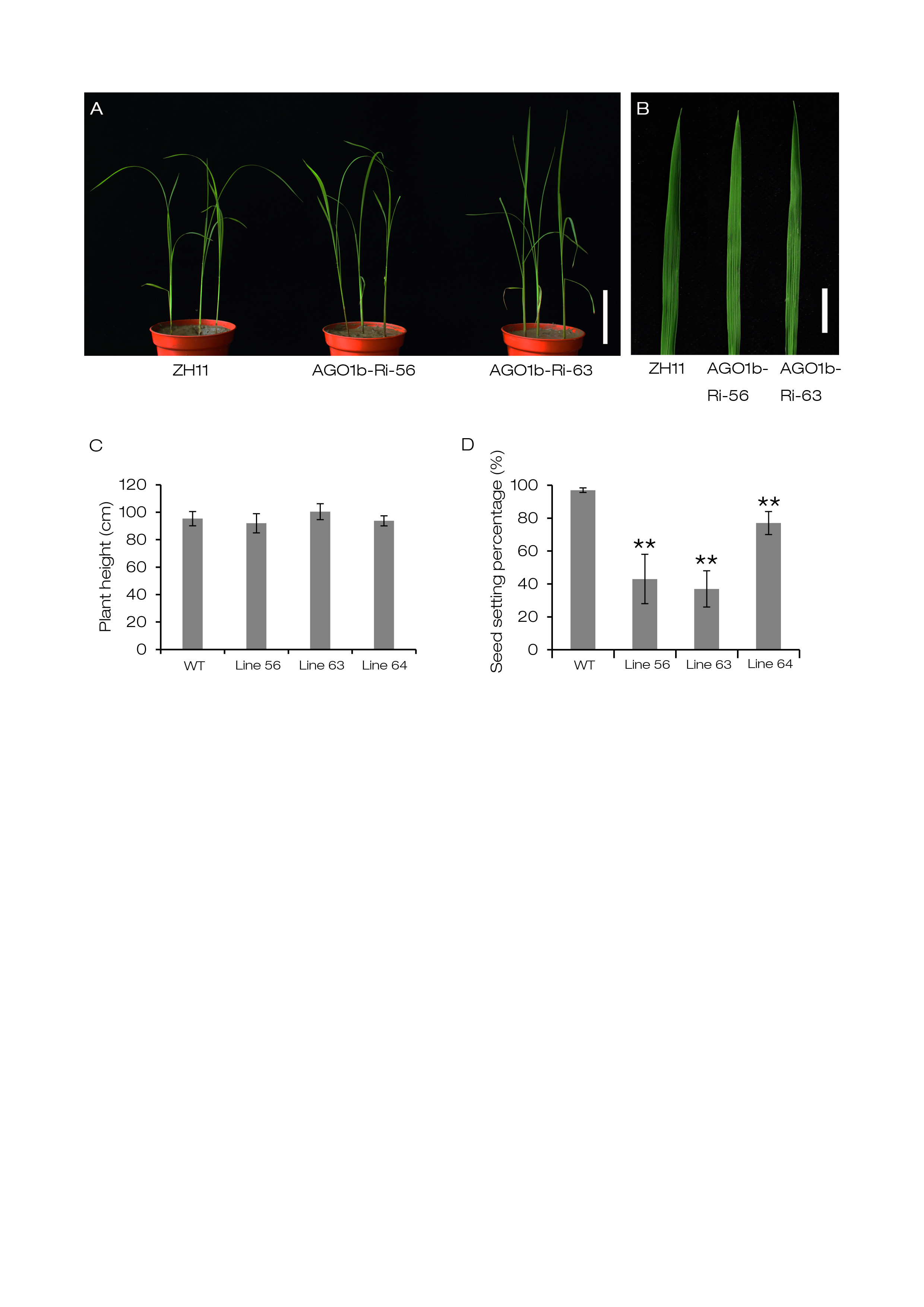

Supplement: Supplementary file 10 — Figure S7. Phenotypes and agronomic traits of ZH11 and OsAGO1b RNAi lines. (A) Phenotypes of 30-day-old seedlings of ZH11 and OsAGO1b RNAi lines, scale bar = 10 cm. (B) The leaves of 30-day-old seedlings of ZH11 and OsAGO1b RNAi lines, scale bar = 3 cm. (C and D) Plant heights and seed setting rates of ZH11 (WT) and OsAGO1b RNAi lines. Results are shown as the mean ± SD in (C)-(D) (n = 10). * P < 0.05, ** P < 0.01 (one-way ANOVA). (TIF 5956 kb) [file 12284_2019_323_MOESM10_ESM.tif]

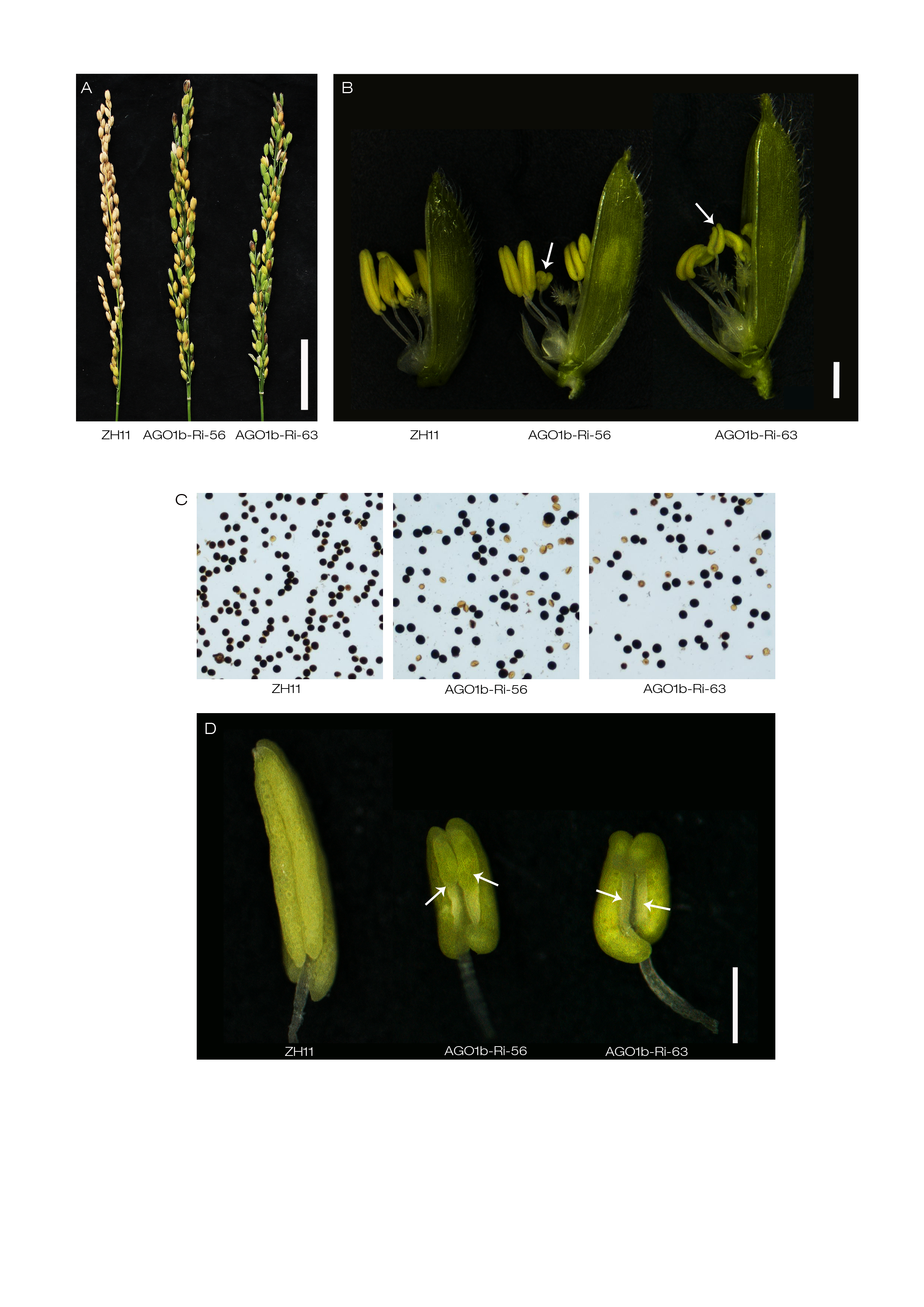

Supplement: Supplementary file 11 — Figure S8. Phenotypes of spikelets and anthers of ZH11 and OsAGO1b RNAi lines. (A) The panicles of ripeness stage, scale bar = 4 cm. (B) Dissection of mature spikelets, the white arrows indicated the curly anthers, scale bar = 1 mm. (C) I2-KI staining of pollens. (D) Phenotypes of anthers, the white arrows indicated the aberrant anther sacs, scale bar = 0.5 mm. (TIF 14943 kb) [file 12284_2019_323_MOESM11_ESM.tif]

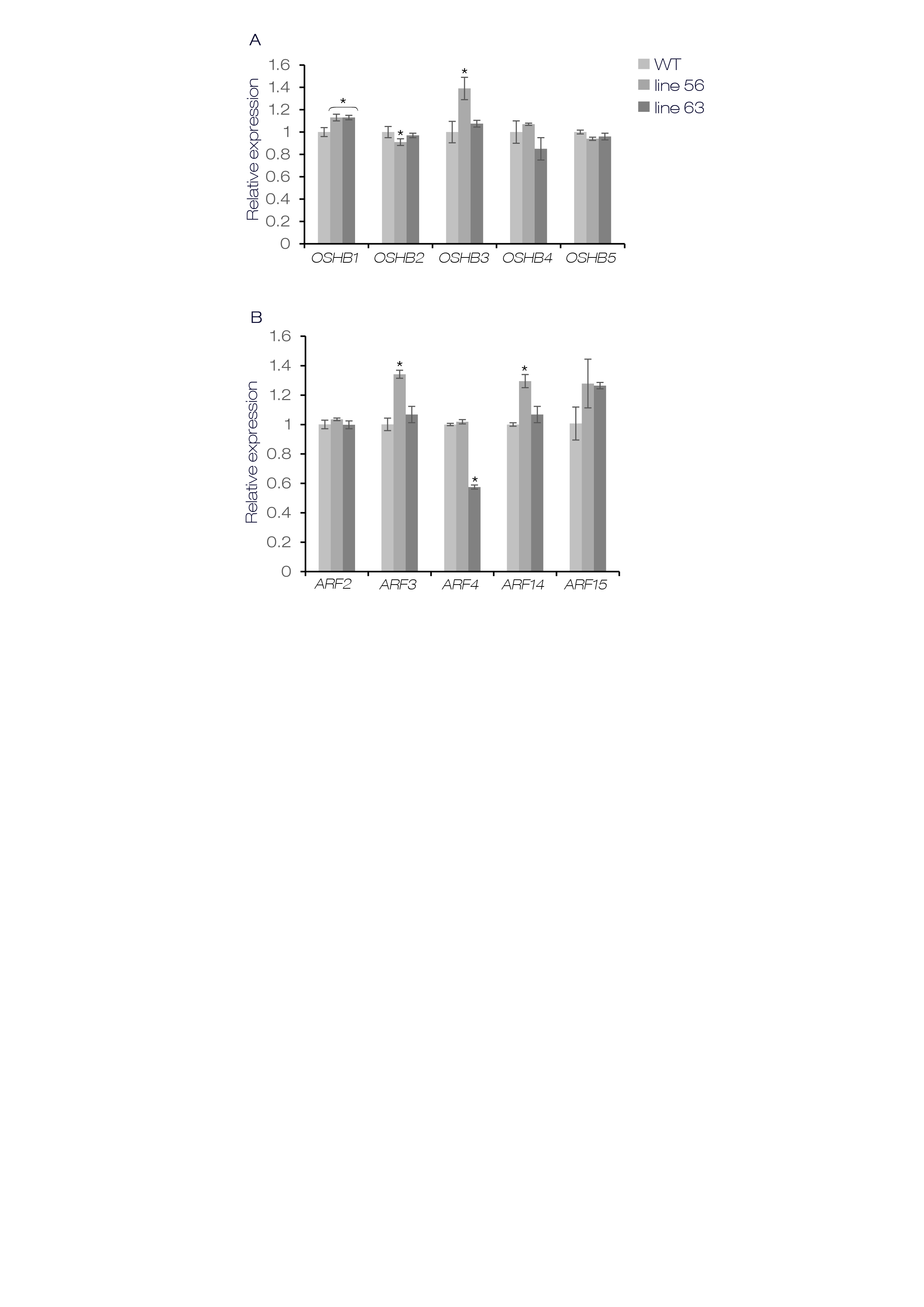

Supplement: Supplementary file 12 — Figure S9. Relative expression of OSHB and OsARF genes in WT and OsAGO1b RNAi lines. The fourth leaves of 30-day-old seedlings from wild-type Zhonghua 11 (WT) and OsAGO1b RNAi line 56 and line 63 were used. The expression level of each gene was normalized to 1 in WT. OsActin1 (XM_015774830) was used as an internal standard to normalize the expression levels of detected genes. Error bars represent standard deviations among replicates (n = 3). * P < 0.05, ** P < 0.01 (one-way ANOVA). (TIF 1542 kb) [file 12284_2019_323_MOESM12_ESM.tif]

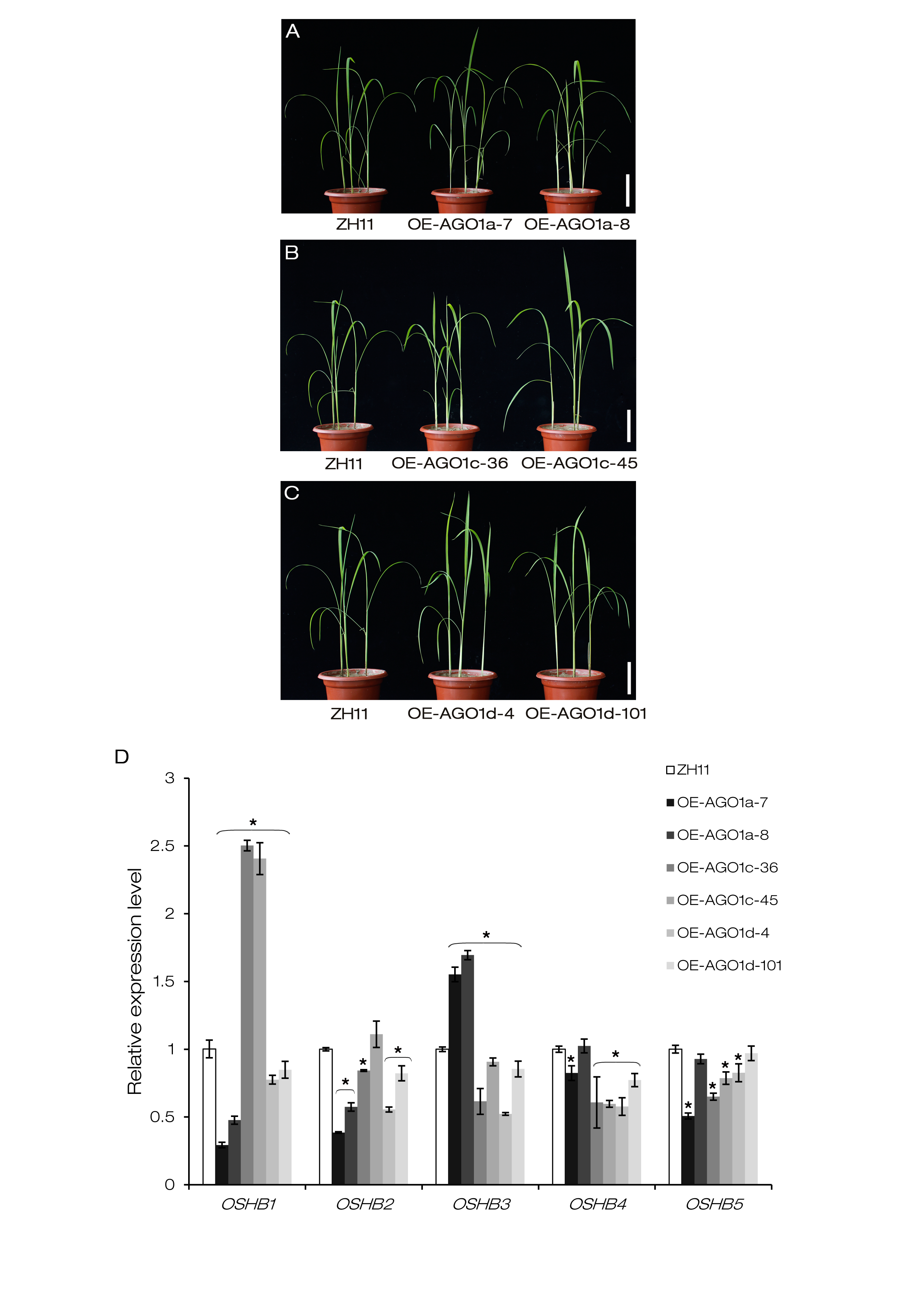

Supplement: Supplementary file 13 — Figure S10. The phenotypes of OsAGO1a, OsAGO1c and OsAGO1d overexpression lines and their expression levels of OSHBs. (A-C) Phenotypes of 30-day-old seedlings of ZH11 and OsAGO1a, OsAGO1c and OsAGO1d overexpression lines, scale bars = 10 cm. (D) Relative expression of OSHBs. The fourth leaves of 30-day-old seedlings from wild-type Zhonghua 11 (ZH11) and each transgenic line were used for RNA extration. The expression level of each gene was normalized to 1 in WT. OsActin1 (XM_015774830) was used as an internal standard to normalize the expression levels of detected genes. OE-AGO1 indicated AGO1 overexprssion lines. Error bars represent standard deviations among replicates (n = 3). * P < 0.05 (one-way ANOVA). (TIF 7387 kb) [file 12284_2019_323_MOESM13_ESM.tif]

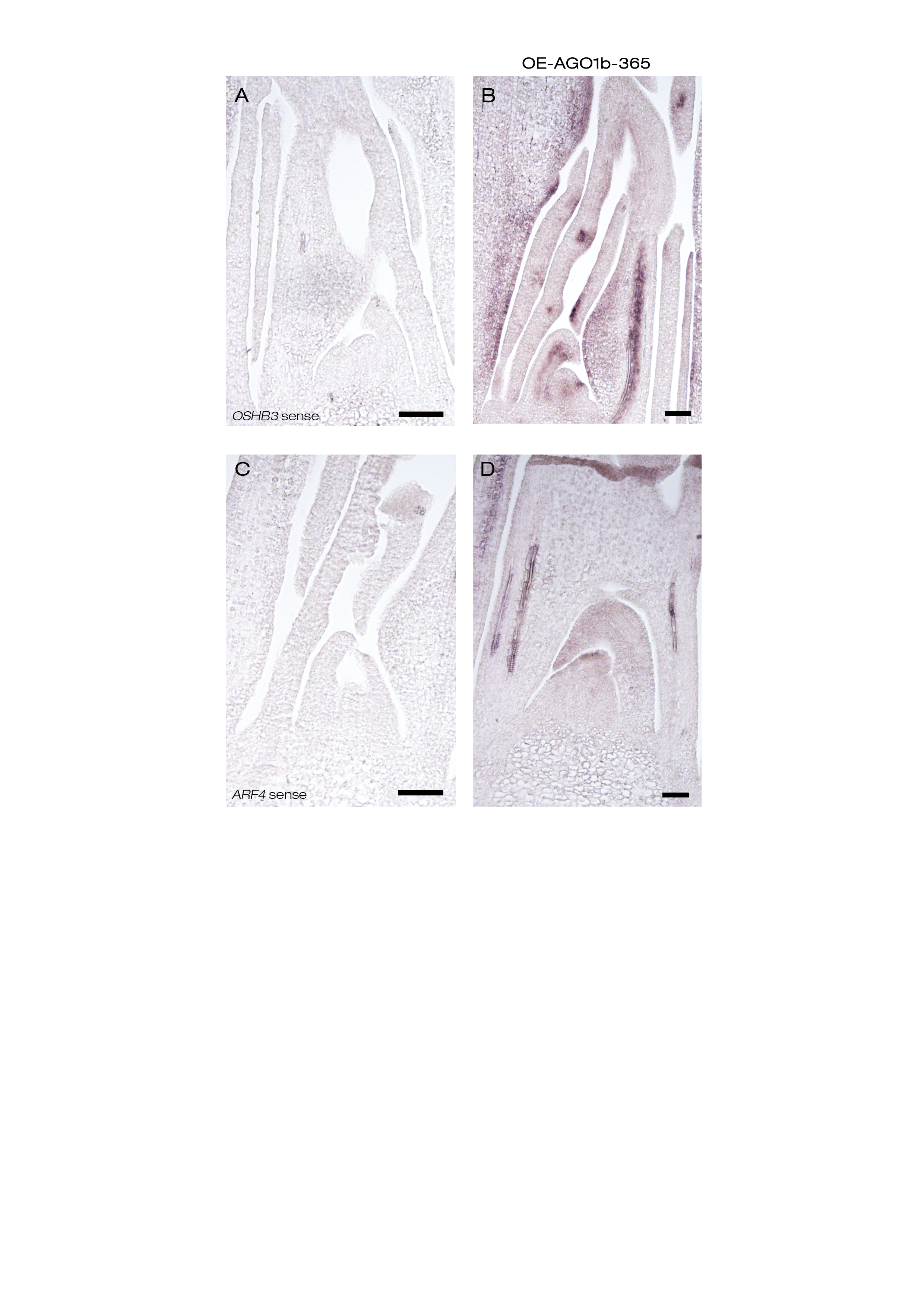

Supplement: Supplementary file 14 — Figure S11. In situ hybridization of OSHB3 and OsARF4 in the vegetative shoot apex of ZH11 and OE-AGO1b-365 (longitudinal sections). (A and C) In situ hybridization of OSHB3 and OsARF4 in ZH11 using sense probe. (B and D) In situ hybridization of OSHB3 and OsARF4 in OE-AGO1b-365 using anti-sense probe. Shoot base cuttings about 1 cm in length including SAM of 14-day-old seedlings were used. Scale bars = 50 μm. (TIF 12330 kb) [file 12284_2019_323_MOESM14_ESM.tif]

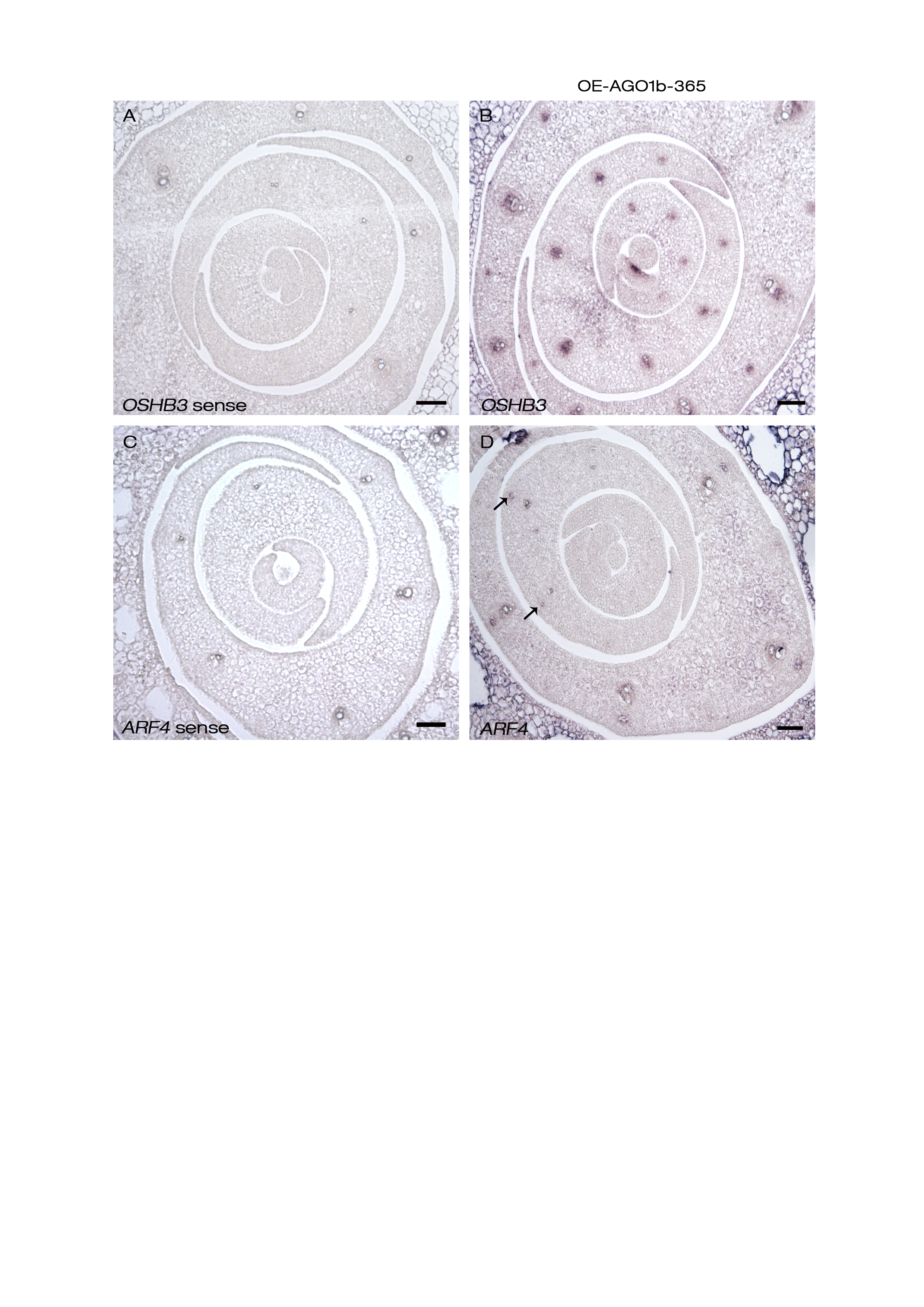

Supplement: Supplementary file 15 — Figure S12. In situ hybridization of OSHB3 and OsARF4 in the vegetative shoot apex of ZH11 and OE-AGO1b-365 (cross sections). (A and C) In situ hybridization of OSHB3 and OsARF4 in ZH11 using sense probe. (B and D) In situ hybridization of OSHB3 and OsARF4 in OE-AGO1b-365 using anti-sense probe. Shoot base cuttings about 1 cm in length including SAM of 14-day-old seedlings were used. Black arrows indicate the developing vasculars. Scale bars = 50 μm. (TIF 16952 kb) [file 12284_2019_323_MOESM15_ESM.tif]
